# Risk of incident cardiovascular disease in people with periodontal disease: A systematic review and meta‐analysis

**DOI:** 10.1002/cre2.336

**Published:** 2020-10-30

**Authors:** Harriet Larvin, Jing Kang, Vishal R. Aggarwal, Sue Pavitt, Jianhua Wu

**Affiliations:** ^1^ School of Dentistry University of Leeds Leeds UK; ^2^ Oral Biology, School of Dentistry University of Leeds Leeds UK; ^3^ Leeds Institute for Data Analytics University of Leeds Leeds UK

**Keywords:** cardiovascular disease, meta‐analysis, meta‐regression, periodontal disease, stroke

## Abstract

**Objectives:**

Cardiovascular disease (CVD) is a major cause of mortality; periodontal disease (PD) affects up to 50% of the world's population. Observational evidence has demonstrated association between CVD and PD. Absent from the literature is a systematic review and meta‐analysis of longitudinal cohort studies quantifying CVD risk in PD populations compared to non‐PD populations. To examine the risk of incident CVD in people with PD in randomised controlled trials and longitudinal cohort studies.

**Material and Methods:**

We searched Medline, EMBASE and Cochrane databases up to 9th Oct 2019 using keywords and MeSH headings using the following concepts: PD, CVD, longitudinal and RCT study design. CVD outcomes included but were not restricted to any CVD, myocardial infarction, coronary heart disease (CHD) and stroke. Diagnosis method and severity of PD were measured either clinically or by self‐report. Studies comparing incident CVD in PD and non‐PD populations were included. Meta‐analysis and meta‐regression was performed to determine risk of CVD in PD populations and examine the effects of PD diagnosis method, PD severity, gender and study region.

**Results:**

Thirty‐two longitudinal cohort studies were included after full text screening; 30 were eligible for meta‐analysis. The risk of CVD was significantly higher in PD compared to non‐PD (relative risk [RR]: 1.20, 95% CI: 1.14–1.26). CVD risk did not differ between clinical or self‐reported PD diagnosis (RR = 0.97, 95% CI: 0.87–1.07,). CVD risk was higher in men (RR: 1.16, 95% CI: 1.08–1.25) and severe PD (RR: 1.25, 95% CI: 1.15–1.35). Among all types of CVD, the risk of stroke was highest (RR = 1.24; 95% CI:1.12–1.38), the risk of CHD was also increased (RR = 1.14; 95% CI:1.08–1.21).

**Conclusion:**

This study demonstrated modest but consistently increased risk of CVD in PD populations. Higher CVD risk in men and people with severe PD suggests population‐targeted interventions could be beneficial.

## 1 INTRODUCTION

Cardiovascular diseases (CVD) are among the leading causes of mortality generating severe burden on global healthcare (Mahase, 2019); while periodontal disease (PD) also affects about 20–50% of the global population and PD prevalence has remained consistently high over the past two decades (Kassebaum et al., 2014). Observational studies have shown an association between periodontal disease (PD) and cardiovascular diseases (CVD). For example, a large cross‐sectional analysis of a cohort of 60,174 people in Norway demonstrated odds ratio of 2.52 for developing atherosclerotic diseases in people with periodontitis (Beukers, van der Heijden, van Wijk, & Loos, 2017). Increased risk of CVD mortality has also been linked to men aged 30–64 years with severe PD (adjusted hazard ratio = 2.13), inflammatory marker levels in men with PD were also significantly higher than those without in this cohort (Xu & Lu, 2011).

Causal links between PD and CVD have previously been postulated. These investigations often implicate PD‐associated bacteria including *Porphyromonas gingivalis* and *Aggregatibacter actinomycetemcomitans* and their associated pathogens and inflammatory pathways as the cause (Bale, Doneen, & Vigerust, 2017; de Boer et al., 2014; Gao et al., 2016; Liao et al., 2009; Mougeot et al., 2017; Sayehmiri et al., 2015). Socransky, Haffajee, Cugini, Smith, and Kent Jr. (1998) suggested endotoxins sourced from the oral microbiota can pathogenically disseminate throughout the body while other findings indicate that *P. gingivalis* may be transported around the body by erythrocytes (Belstrom et al., 2011; Socransky et al., 1998). Pathogenic and systemic effects of bacteraemia have also been observed in patients after oral procedures (Baltch et al., 1988), advocating an additional possible causal association. While there is still much to be learnt regarding the actual mechanism between PD and subsequent CVD, the data linkage of electronic dental, medical and insurance records permits non‐intrusive observational research into associations between CVD and PD (Mark Bartold & Mariotti, 2017).

Previous reviews have explored the relationship between PD and CVD conditions. Liccardo et al. (2019) qualitatively reviewed the evidence for associations between PD and CVD (Liccardo et al., 2019). Furthermore, in a recent meta‐analysis Aguilera et al. (2019) found patients with periodontitis are 1.68 times more likely to develop hypertension (Aguilera et al., 2019). PD is also found to be associated with peripheral artery disease (PAD), stroke and coronary heart disease (CHD) (Dietrich, Sharma, Walter, Weston, & Beck, 2013; Martin‐Cabezas et al., 2016; Yang et al., 2018). These reviews often incorporate the more widely available cross‐sectional studies which can provide prevalence estimates but do not necessarily demonstrate the potential causal link between PD and CVD (Slots, 1998).

Absent from the literature is a meta‐analysis of longitudinal cohort studies quantifying the risk of CVD in PD populations compared to non‐PD populations. Furthermore, as observational studies can be highly heterogenous, meta‐regression alongside a meta‐analysis could allow the exploration of risk factors and study characteristics and the effect to CVD risk.

The aim of the current investigation was to conduct a systematic review to examine the risk of incident CVD outcomes in people with PD compared to non‐PD. In adhering to this aim, we sought to conduct a meta‐analysis of studies that quantify risk of CVD in PD populations, with additional meta‐regression to evaluate the impact of key risk factors. Full appraisal of the evidence quantifying this directional association will improve the understanding of PD prognosis and highlight the requirement of improved oral health practice and targeted chronic CVD prevention initiatives.

## 2 METHODS

Study design—a systematic review of randomised controlled trials and longitudinal cohort studies that examine the risk of CVD in people with PD compared to a non‐PD populations.

### 2.1 Search strategy

The search string considered alternate terms and a variety of CVD outcomes incorporating several relevant key words and Medical Subject Headings (MeSH) headings. The final Boolean search string was: (periodon* OR tooth loss OR missing teeth) AND (atrial fibrillation OR heart failure OR cerebrovascular accident OR stroke OR angina OR acute coronary syndrome OR myocardial infarction OR peripheral vascular disease OR hypertension OR cardiovascular disease) AND (incidence) AND (cohort OR longitudinal OR randomi*ed controlled trial OR RCT) (Table S1).

The search string was applied from database conception until 9th October 2019 to Medline, EMBASE and Cochrane databases to ensure retrieval of a broad scope of literature. Additional reference checking and “citation snowballing” methods of key articles were also undertaken to maximise search sensitivity.

### 2.2 Study selection

Following database searches, retrieved studies were imported into a citation manager and screened for duplicates using an automated system. One author screened for title and abstract for eligibility with validation by a second author. Whole articles were examined for eligibility before conducting data extraction and quality assessment. Consensus for included studies was made between the authors and disagreements were resolved by thorough discussion. A data extraction form was developed prior to the database search in order to identify key study information including population demographics, data source, exclusion criteria, follow up period, outcome measure and limitations of the study. Results from data extraction were monitored by a second author and queries were discussed at length to ensure adherence to the protocol.

### 2.3 Eligibility criteria

Strict eligibility criteria guided the search to ensure relevant study inclusion, reduce heterogeneity and increase power of results. The inclusion criteria were outlined as the following:

- Longitudinal retrospective/prospective cohort and randomised controlled trials study design.
  - PD population free from predefined systemic disease at baseline.
  - Minimum of 1‐year follow‐up period.
  - Clinically diagnosed or self‐reported PD.
  - Clearly defined classification of CVDs.
  - Peer reviewed articles and published in English.

Exclusion from the review was elicited if the study fell under one of the following criteria:

- Cross‐sectional, case‐series or case–control study.
- Animal studies or studies on populations with predefined systemic disease prior to follow‐up.
- Protocols, abstracts, reviews or conference proceedings.
- Lack of validated or clearly defined diagnosis of PD.
- Absent or unclear definition of CVD.
- Risk of CVD not accessible.

The minimum follow‐up period was 1 year following PD diagnosis and populations with pre‐diagnosed CVD conditions were not eligible to ensure accurate CVD incidence calculation, rather than prevalence of an undiagnosed condition that may have preceded PD. Clinical PD classifications comprised clinical examination or the identification of appropriate ICD‐9/10 codes within electronic health records or insurance databases. Questionnaire or interview responses were classified as self‐reported PD. Evaluation of dental hygiene, presence of dental caries, cysts or lesions and other conditions such as gingivitis, peri‐implantitis and odontogenic infection were not accepted as case definitions for PD as they are not directly attributed to PD (British Society of Periodontology, 2016).

### 2.4 Quality assessment

Quality assessment tools for observational studies can be contentious (Sanderson, Tatt, & Higgins, 2007), therefore this review employed the Risk of Bias in Non‐Randomised Studies of Interventions (ROBINS‐I) recommended by Cochrane to determine risk of bias in cohort and longitudinal observational studies (Sterne et al., 2016). Results from the risk of bias assessment were conferred with a second author and discrepancies discussed before finalising ROBINS‐I assessment table.

The review protocol was pre‐registered to the PROSPERO database before the study began (registration number: CRD42019154897).

### 2.5 Statistical analysis

Odds ratios (OR), hazard ratios (HR) and relative risks (RR) were used in different studies to quantify the risk of CVD. ORs and HRs were converted into RRs according to Shor, Roelfs, and Vang (2017) to increase the study size (Shor et al., 2017). The adjusted RRs were used to pool for meta‐analysis and adjustments of key confounders, such as smoking, gender and age, were screened for each study. For inclusion in meta‐analysis, studies must have reported population numbers for PD and non‐PD and RRs or converted RRs should also be available to be eligible for synthesis and pooling. Random effect meta‐analysis was performed for the overall CVD outcome and separately for each individual CVD outcomes given that there were more than five studies reporting the same CVD outcome (Viechtbauer, 2010). Subgroup analysis was performed for PD diagnosis method, PD severity, gender and study regions. Meta‐regression was conducted to compare the risk of CVD between the subgroups (Viechtbauer, 2010). *I*^2^ was used to measure the study heterogeneity.

Potential publication bias was depicted by funnel plots and was quantified by Egger's test. Forest plots were used to visualise the pooled results for the overall risk of CVD and the individual CVD outcomes (Higgins et al., 2019). Sensitivity analysis was conducted to assess the effect of the study characteristics on the risk of CVD. For those included studies not eligible for meta‐analysis, key study features were narrated in the results.

## 3 REVIEW

**FIGURE 1:**
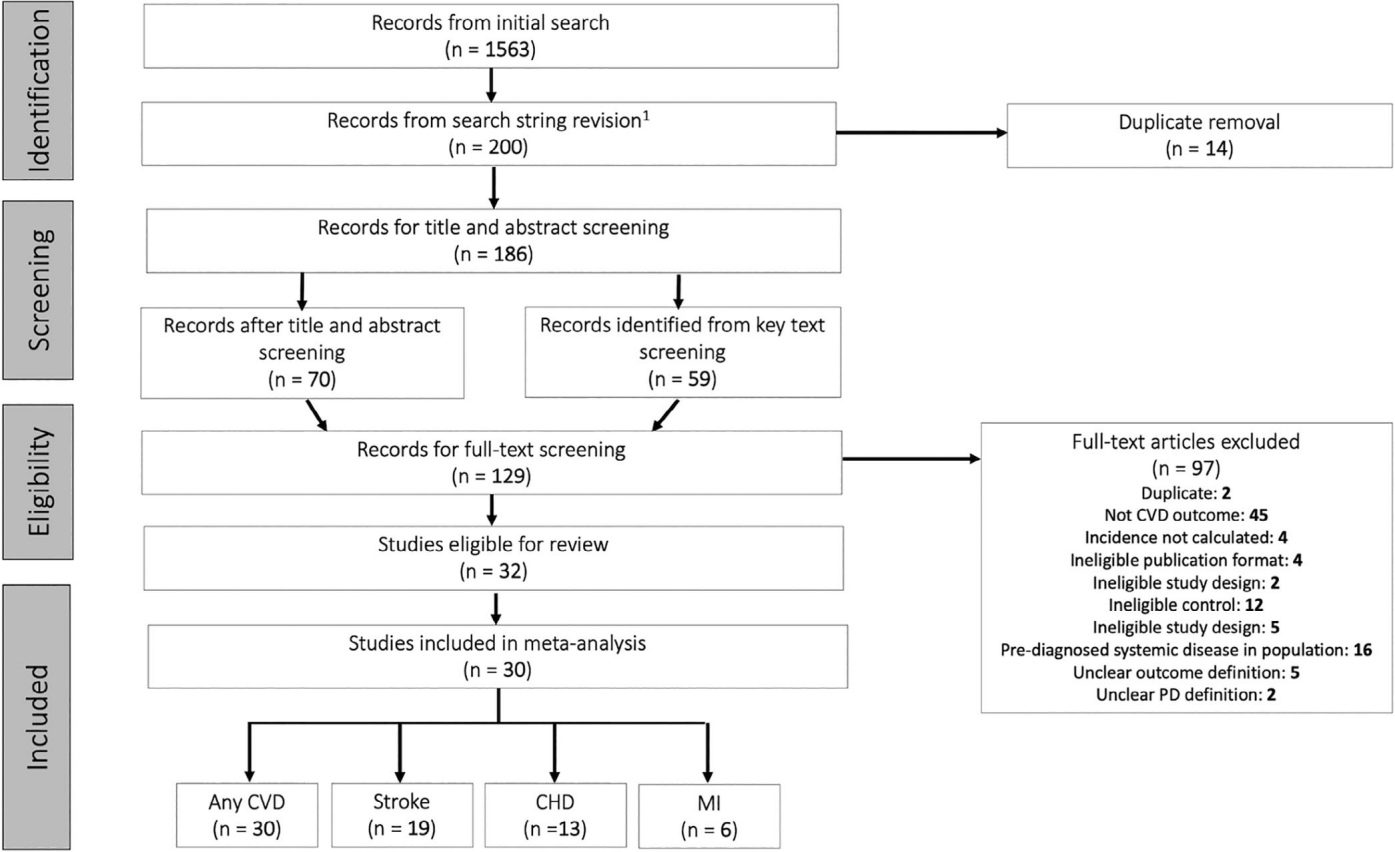
PRISMA flow chart demonstrating search strategy. *Note:* coronary heart disease (CHD), cardiovascular disease (CVD), myocardial infarction (MI), number of studies (*n*), periodontal disease (PD)

A total of 1,563 studies were retrieved from the initial search. After screening, 129 studies were screened for the full content, and 32 studies were included (PRISMA flow chart, Figure 1). Two studies were excluded from meta‐analysis as raw data concerning sample sizes could not be extracted (Abnet et al., 2005; Holmlund, Lampa, & Lind, 2017).

Included studies were published between 1993 and 2019 and are fully described in the supplementary file (Table S2). Most studies were based in United States (*n* = 16), the remaining studies used data from developed countries across Europe, Asia and Australia. Median follow up period was 14.5 years (Interquartile range: 10.0–20.3 years). Of the 32 included studies, 22 of them were prospective cohort studies, and 21 of them used cohorts with clinically diagnosed PD (Table 1). Fourteen studies examined the risk of CVD in men, while six studies reported CVD risk in women. All studies reported age‐adjusted risk of CVD however five studies did not adjust for smoking. See Table S2 for more details.

**TABLE 1:** Summary table of included studies, where conditions were not specified as stroke/CHDMI, these are classified as any CVD

Study	Study design	Total population	Location	PD diagnosis	Outcome	Total study follow‐up period (years)	Risk of bias
Abnet et al., 2005 ^b^	Prospective	29,584	China	Clinical	Stroke	15	Critical
Batty et al., 2018	Prospective	626,106	Korea	Clinical	CHD	21	Serious
Beck et al., 1996	Prospective	1,147	USA	Clinical	CHD + stroke	18	Critical
Chen, Lin, Chen, & Chen, 2016	Retrospective	787,490	Taiwan	Clinical	AF	10	Critical
Choe et al., 2009	Prospective	679,170	Korea	Clinical	Stroke	14	Critical
Chou et al., 2015 ^a^	Retrospective	27,146	Taiwan	Clinical	Any CVD	9	Critical
DeStefano, Anda, Kahn, Williamson, & Russell, 1993 ^a^	Prospective	9,760	USA	Clinical	CHD	16	Serious
Dietrich et al.,^2008^	Prospective	1,203	USA	Clinical	CHD	35	Critical
Hansen, Egeberg, Holmstrup, & Hansen, 2016	Prospective	100,694	Denmark	Clinical	Stroke + MI	15	Critical
Heitmann & Gambourg, 2008	Prospective	2,932	Denmark	Clinical	CHD	7	Serious
Holmlund et al., 2017 ^b^	Prospective	8,999	Sweden	Clinical	Any CVD	34	Critical
Howell, Ridker, Ajani, Hennekens, & Christen, 2001	RCT	22,037	USA	Self‐report	Stroke + MI	13	Critical
Hujoel, Drangsholt, Spiekerman, & DeRouen, 2001	Prospective	8,032	USA	Clinical	CHD	20	Serious
Hung et al., 2003	Prospective	45,094	USA	Self‐report	PAD	12	Critical
Hung et al., 2004	Retrospective	100,381	USA	Self‐report	CHD	12	Critical
Jimenez et al., 2009	Prospective	1,231	USA	Clinical	Stroke	34	Critical
Joshipura 1996b	Prospective	44,119	USA	Self‐report	CHD	6	Critical
Joshipura, Hung, Rimm, Willett, & Ascherio, 2003	Prospective	41,380	USA	Self‐report	Stroke	12	Critical
Joshy et al., 2016	Prospective	172,630	Australia	Self‐report	CHD + stroke	5	Serious
LaMonte et al., 2017	Prospective	57,001	USA	Self‐report	CHD + stroke	12	Critical
Lee et al., 2013 ^a^	Retrospective	719,436	Taiwan	Clinical	Stroke	10	Critical
Lee, Hu, Chou, & Chu, 2015 ^a^	Retrospective	720,343	Taiwan	Clinical	MI	10	Critical
Lee et al., 2017	Retrospective	354,850	South Korea	Clinical	Stroke + MI	12	Critical
Lin et al., 2019	Retrospective	161,923	Taiwan	Clinical	Stroke	10	Critical
Morrison, Ellison, & Taylor, 1999	Retrospective	9,331	Canada	Clinical	CHD + stroke	23	Serious
Mucci et al., 2009	Prospective	15,273	Sweden	Self‐report	CHD + stroke	37	Serious
Noguchi et al.,^2014^	Prospective	3,081	Japan	Self‐report	MI	5	Serious
Rivas‐Tumanyan, Spiegelman, Curhan, Forman, & Joshipura, 2012	Prospective	31,543	USA	Self‐report	Hypertension	20	Critical
Sen et al., 2018	Retrospective	6,736	USA	Clinical	Stroke	15	Serious
Tu et al., 2007	Prospective	12,631	UK	Clinical	CHD + stroke	57	Serious
Wu et al., 2000	Prospective	9,962	USA	Clinical	Stroke	22	Serious
Yu et al., 2015	Prospective	39,863	USA	Self‐report	Stroke + MI	16	Critical

**Abbreviations:** AF, atrial fibrillation; CHD, coronary heart disease; CVD, cardiovascular disease; MI, myocardial infarction; *n*, number of studies; PAD, peripheral artery disease; PD, periodontal disease; RCT, randomised controlled trial.

^a^ Studies that did not adjust for smoking.

^b^ Study not included in meta‐analysis.

According to ROBINS‐I assessment, 21 studies were categorised as critical risk of bias, and the remaining 11 were at serious risk (Table S3). There was significant risk of publication bias as demonstrated in a funnel plot (Figure S1) and by Egger's test (Egger's test: *β =* 2.91, *p* = .004).

**FIGURE 2:**
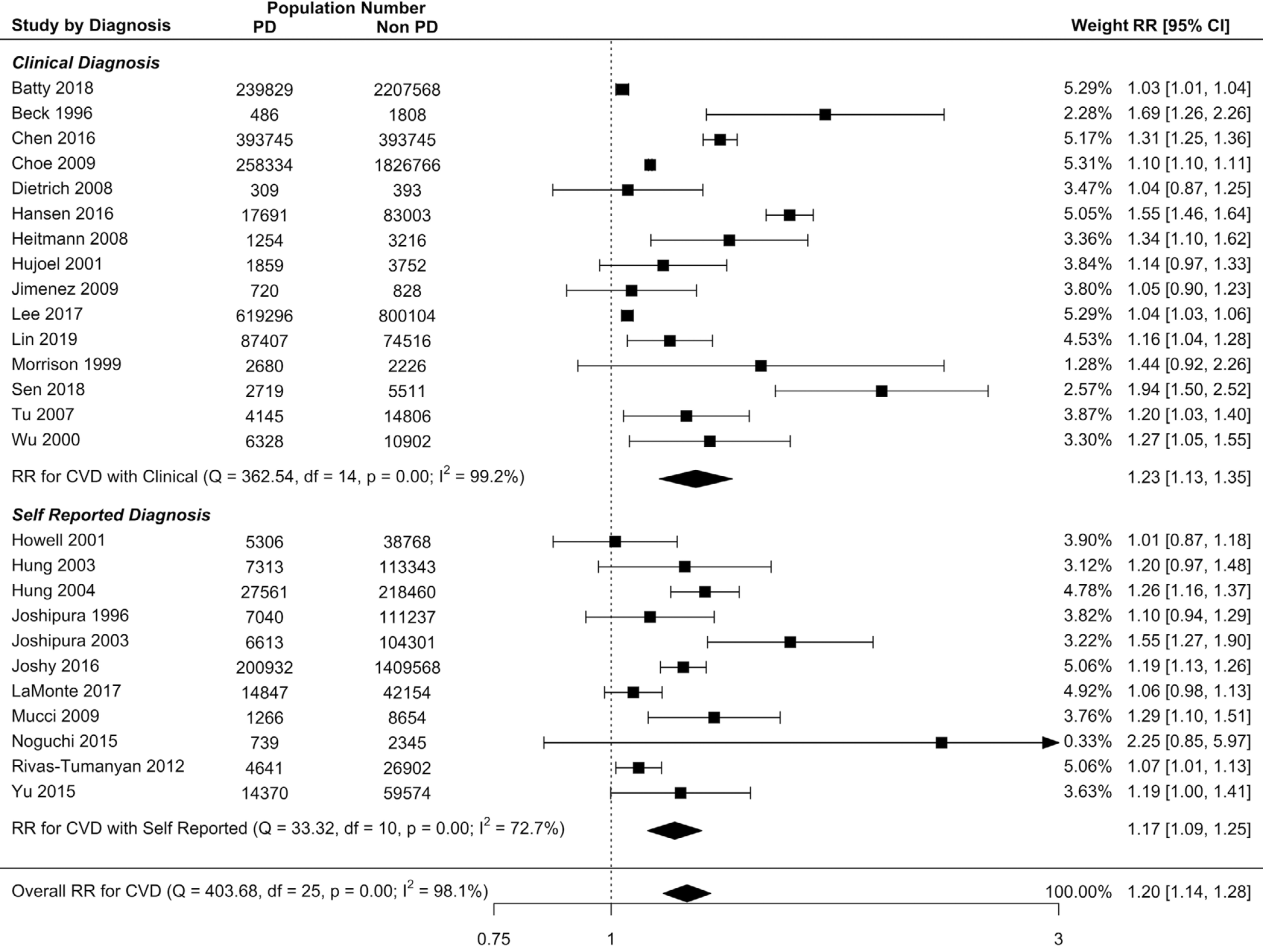
Forest plot illustrating results from random effect meta‐analysis for the incident risk of all CVD in people with PD and by PD diagnosis method

Overall, random effects meta‐analysis demonstrates that there is a significant increase in risk of all incident CVD in PD population compared to non‐PD population (RR = 1.20, 95% CI: 1.14–1.26; Figure 2). There was quite high heterogeneity (*I*^2^ = 97.3%) due to the large sample size in most studies. For individual CVD outcomes with more than six reporting studies, the risk of stroke in PD increased by 24% in PD (RR = 1.24, 95% CI: 1.12 to 1.38; Figure S2), and the risk of CHD increased by 14% (RR = 1.14, 95% CI: 1.08–1.21; Figure S3). While there was an increased risk of MI, the precision of the estimate based on the confidence interval bounds was not significant (RR = 1.12, 95% CI: 0.96–1.30; Figure S4).

Two studies were not included in meta‐analysis as study numbers of PD and non‐PD were not available. Holmlund et al. (2017) found increased risk of all incident CVD using clinical PD diagnosis (RR = 1.18, 95% CI: 1.01 to 1.23) (Holmlund et al., 2017). Abnet et al. (2005) also published increased risk of stroke in people with more tooth loss (RR = 1.11, 95% CI: 1.01–1.23) (Abnet et al., 2005).

**FIGURE 3:**
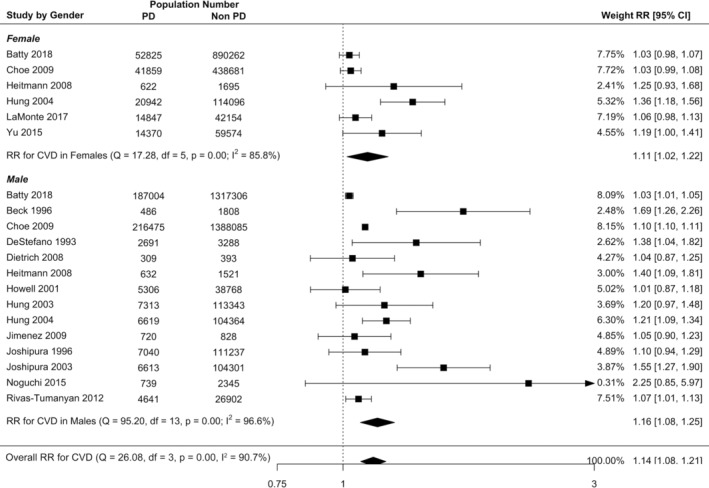
Forest plot illustrating results from random effect meta‐analysis for the incident risk of all CVD in people with PD and by gender subgroup

Subgroup meta‐analysis revealed that the risk of all incident CVD in studies utilising clinical PD diagnosis was 22% higher in PD (RR = 1.22, 95% CI: 1.14–1.30) and in self‐reported diagnosis was 17% higher in PD (RR = 1.17, 95% CI: 1.09–1.25) compared with non‐PD (Figure 2), and there was no significant difference in diagnosis method using meta‐regression (RR = 0.97, 95% CI: 0.87–1.07, Table 2). The incident risk of CVD was 16% higher in men with PD (RR = 1.16, 95% CI: 1.08–1.25) and 11% higher in women with PD (RR = 1.11, 95% CI: 1.02–1.22) compared with non‐PD (Figure 3), however the CVD risk was not significantly different between male and female (RR = 1.04, 95% CI: 0.92–1.17).

**TABLE 2:** Results from meta‐regression models demonstrating between‐group difference of PD diagnosis, gender, PD severity and region as independent variables

Variable	*n*	Risk ratio (95% CI)
**Diagnosis method**		
Clinical	19	1
Self‐reported	11	0.97 (0.87–1.07)
**Gender**		
Female	6	1
Male	14	1.04 (0.92–1.17)
**PD severity**		
Mild	12	1
Moderate	15	1.10 (0.99–1.22)
Severe	18	1.11 (1.00–1.22)
**Region**		
Asia/Australia	10	1
Europe	4	1.18 (1.03–1.35)
North America	16	1.03 (0.93–1.13)

Abbreviations: CI, confidence intervals; PD, periodontal disease; *n*, number of studies.

**FIGURE 4:**
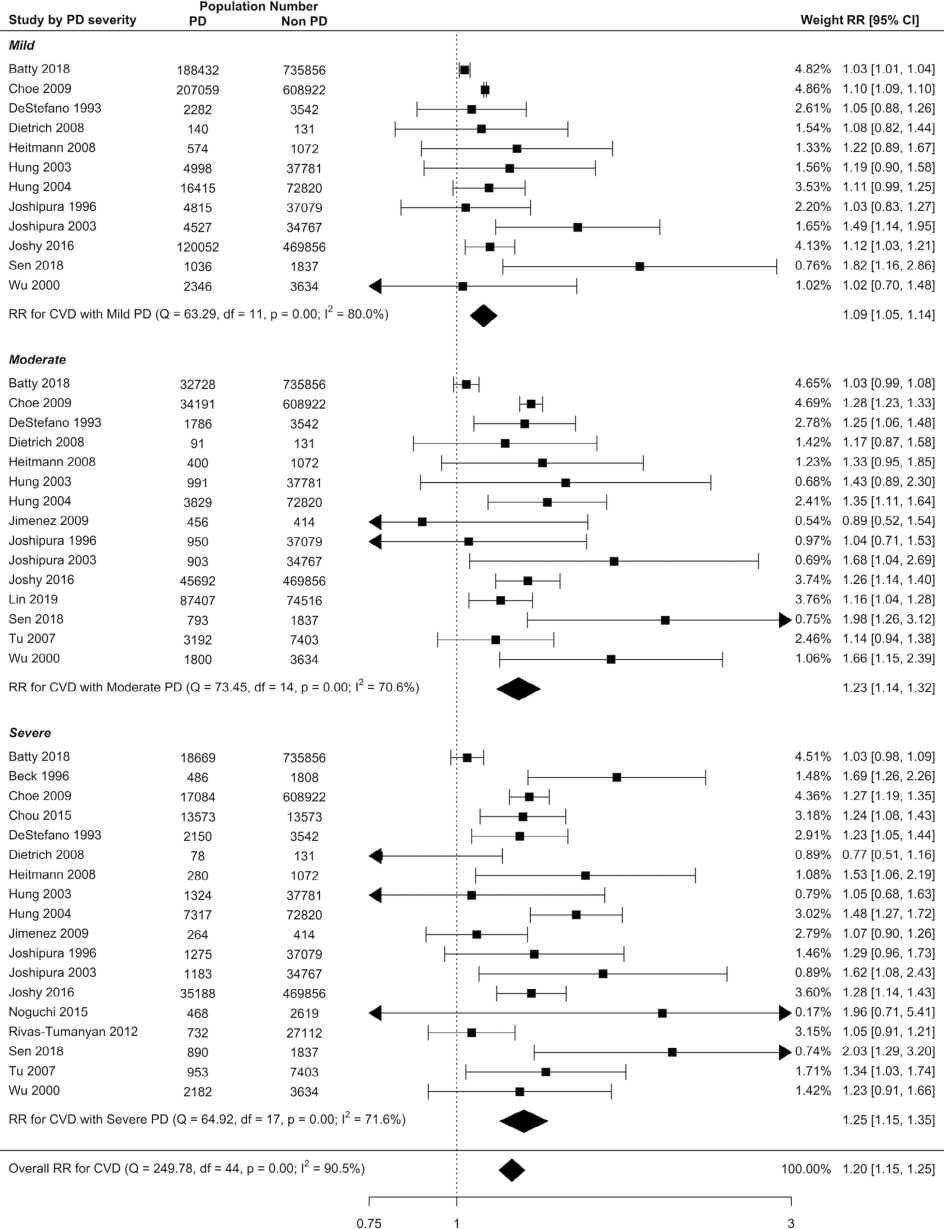
Forest plot illustrating results from random effect meta‐analysis for the incident risk of all CVD in people with PD and by PD severity subgroup

The risk of incident CVD increased ascendingly with the PD severity (Figure 4) from 9% for mild PD (RR = 1.09, 95% CI: 1.05–1.14), to 23% for moderate PD (RR = 1.23, 95% CI: 1.14–1.32) and 25% for severe PD (RR = 1.25, 95% CI: 1.15–1.35). The risk of incident CVD was significantly higher in severe PD compared with mild PD (RR = 1.11, 95% CI: 1.00–1.22; Table 2).

**FIGURE 5:**
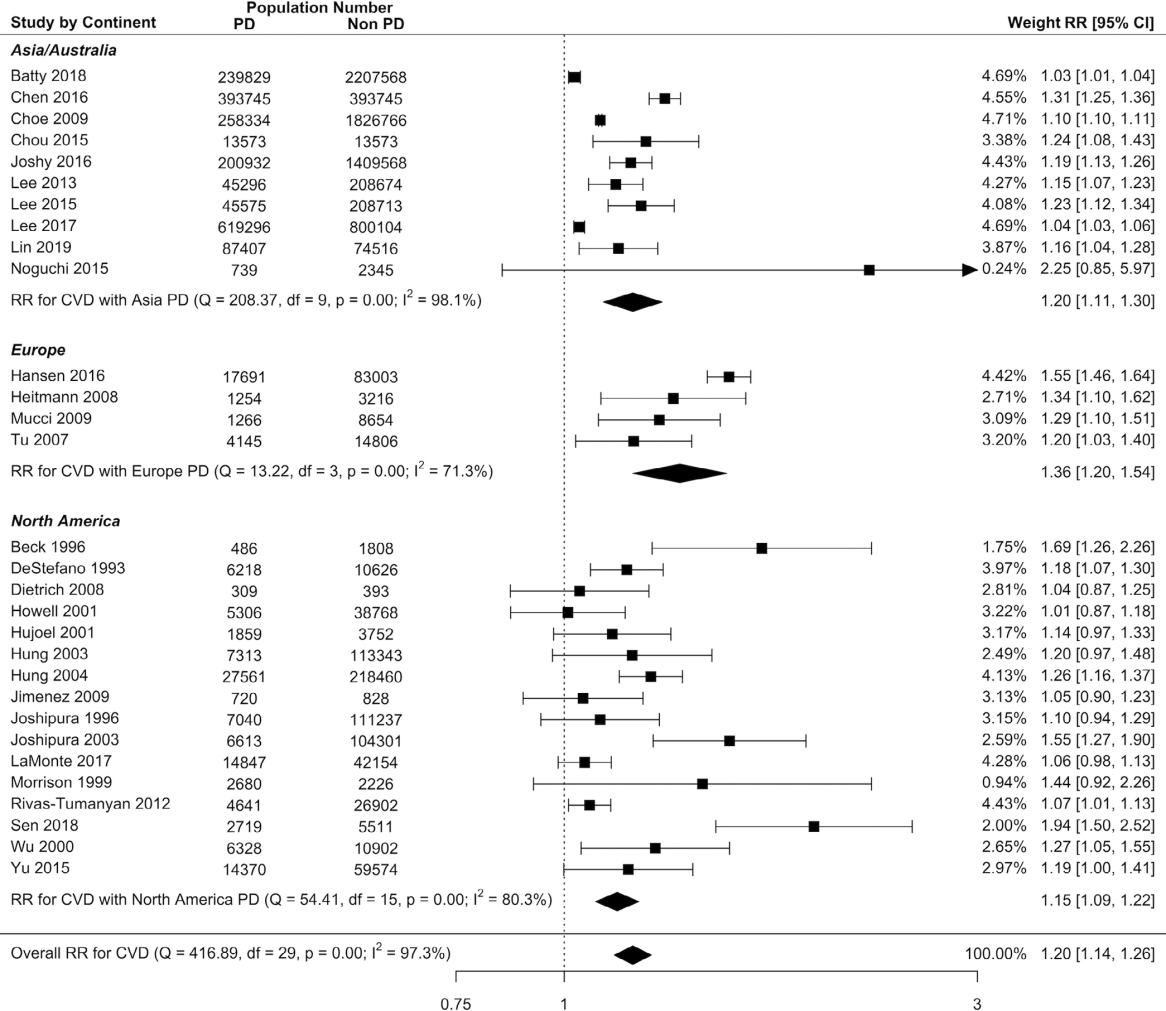
Forest plot illustrating results from random effect meta‐analysis for the incident risk of all CVD in people with PD and by geographical region subgroup

Subgroup analysis by geographic regions demonstrated that the incident CVD risk in European studies (RR = 1.36, 95% CI: 1.20–1.54; Figure 5) was 18% higher (RR = 1.18, 95% CI: 1.03–0.35; Table 2) compared with studies from Asia/Australia, and 3% higher (RR = 1.03, 95% CI: 0.93–1.13; Table 2) compared with studies from North America.

Sensitivity analysis was conducted by excluding five studies without adjusting for smoking, and this did not change the risk of CVD (RR = 1.20, 95% CI: 1.14–1.28; Figure S5). The effect of excluding health professional's self‐reported PD was also examined in a sensitivity analysis. This increased overall risk of CVD (RR = 1.24, 95% CI: 1.14–1.34; Figure S6). Risk of CVD with self‐reported PD in non‐health professionals was 3% lower (RR = 0.97, 95% CI: 0.84–1.13; Table S4) compared to clinical PD diagnosis method.

## 4 DISCUSSION

This systematic review and meta‐analysis examined the risk of incident CVD in people with PD using the longitudinal cohort studies. The findings demonstrate that there is a higher risk of all incident CVD outcomes in PD populations compared to non‐PD, and this risk is consistent across the PD diagnosis method, PD severity, gender and study regions. The risk of incident CVD is no different between clinical and self‐reported diagnosis, or between male and female, but the CVD risk is associated with PD severity and study regions.

The meta‐analysis quantified the risk of incident CVD in people with PD compared with non‐PD. Our findings reflect qualitative conclusions from previous reviews. Dietrich et al. (2013) recounted significantly increased risk of CVD across 11 of 12 studies, however meta‐analysis was not attempted (Dietrich et al., 2013). Scannapieco, Bush, and Paju (2003) also reported moderate positive association between PD and CVD prevalence in a narrative review inclusive of cross‐sectional and case–control studies (Scannapieco et al., 2003). Our study not only quantified the risk of all incident CVD, but also included the risk of incident CHD, stroke, and MI. The finding of increased risk of stroke and CHD have been reflected in previous reviews (Bahekar, Singh, Saha, Molnar, & Arora, 2007; Lafon et al., 2014). The increase in risk of MI was not significant in the present study, as demonstrated by the precision of the estimate according to confidence intervals. This could be due to sample size limitations and also reflects conclusions from previous reviews (Sidhu, 2016; Xu et al., 2017).

Though the association was not quantified, more recently the causal relationship and mechanism between PD and CVD has been explored, suggesting the two diseases are linked by shared inflammatory pathways (Liccardo et al., 2019). Increased platelet count and augmented C‐reactive protein levels are indicators of systemic inflammation that have been observed in both PD and CVD conditions (Linden, McClean, Young, Evans, & Kee, 2008; Romandini, Laforí, Romandini, Baima, & Cordaro, 2018; Sun et al., 2019). Disrupted cell signalling induced by inflammation could induce cell death and result in systemic symptoms such as those observed in CVD conditions (Sun et al., 2019). There have also been findings that suggest PD treatment may alleviate CVD symptoms and inflammation and the association between conditions may be bi‐directional (D'Aiuto et al., 2004; Roca‐Millan et al., 2018).

The present review revealed higher risk of CVD in males with PD compared to females. Though meta‐regression suggested that gender does not affect the risk of CVD, previous research has indicated that hormonal gender differences may result in different systemic pathologies between males and females (Marchetti et al., 2012; Nazir, 2017). Further research should include gender as a separate risk factor for PD in order to explore this association.

Our finding of most increased CVD risk in severe PD has been reflected in other systematic reviews (Dietrich et al., 2013; Martin‐Cabezas et al., 2016), however direct comparisons of findings cannot be reliably made as these reviews did not produce meta‐analysis or consider risk of CVD in mild PD as a comparator, respectively. Our study has demonstrated a gradient relationship between PD severity and CVD risk. The higher CVD risk in the severe PD population indicates that potential targeted intervention could be more effective in reducing the CVD risk towards severe PD populations.

The utility of a proxy such as self‐reported as evidence for a diagnosis of PD is controversial. The present review demonstrates little difference in the risk of CVD using a clinical PD definition. Previous research has indicated self‐reported PD prevalence is underestimated in populations (Heloe, 1972; Pitiphat, Garcia, Douglass, & Joshipura, 2002) with suggestions that validity of self‐reported PD may be population and question‐dependent, with higher accuracy in clinicians and for self‐reported severe PD (Blicher, Joshipura, & Eke, 2005; Heaton et al., 2017; Joshipura, Douglass, Garcia, Valachovic, & Willett, 1996a). Given that several included studies utilised data from the Health Professionals Follow Up Study and the Nurses' Health Study (Hung et al., 2003; Hung et al., 2004; Joshipura et al., 1996b; Joshipura et al., 2003; Lee et al., 2017; Rivas‐Tumanyan et al., 2012), we conducted sensitivity analysis to determine the effect of these populations on risk of CVD. The results of the sensitivity analysis did not affect the risk of CVD, suggesting self‐reported PD in health professionals and the general public are viable for pooling in meta‐analysis. Our findings reflect previous research by Abbood, Hinz, Cherukara, and Macfarlane (2016), who concluded validity of PD diagnosis is not compromised when using self‐reported diagnosis as a self‐report (Abbood et al., 2016).

Several included studies did not adjust for smoking, a major modifiable risk factor for PD (Chou et al., 2015; DeStefano et al., 1993; Lee et al., 2017; Lee et al., 2015; Y. L. Lee et al., 2013; Lin et al., 2019). Smoking is also significantly associated with all CVD outcomes (Banks et al., 2019), in addition to increased periodontal tissue attachment loss and destruction of alveolar bone (Gautam et al., 2011). The results of the sensitivity analysis suggest adjustments for smoking as a confounder may not significantly affect estimates for CVD risk in people with PD. Stress is also another potential confounder to both PD and CVD as it can have oral and systemic pathological effects (Hawken et al., 2004; Jebi et al., 2017). This variable was not consistently adjusted in the included studies and therefore we were not able to account for this in the meta‐regression. Further research is required to address the impact of confounding effects further and form robust conclusions.

### 4.1 Strengths and limitations

To our knowledge this meta‐analysis is the first of its kind in this area of research, consequently there are noteworthy strengths. By utilising subgroup and meta‐regression meta‐analysis, we explored the effect of PD diagnosis method, gender, study region, PD severity on subsequent risk of all incident CVD and individual CVD conditions. As such we were able to include more longitudinal studies than before in a systematic review of this topic, and we were able to investigate the impacts of risk factors on multiple CVD outcomes. As only longitudinal and RCT studies were included in this systematic review, we were also able to draw causal inference. This review strictly adhered to PRISMA guidelines which strengthens the reliability of our report.

There are also some limitations to this study. Given that the eligible studies were observational by design there was high risk of bias, confounding effects and heterogeneity across the pooled estimates. We strove to account for this by undertaking full ROBINS‐I assessment to identify risk of bias, conducting subgroup random effects meta‐analysis and meta‐regression, in addition to exploration by sensitivity analysis. These analyses only accounted for some of the heterogeneity observed. Some population demographics such as age could not be measured as full data was not available for all included studies. Previous cross‐sectional research has revealed strong associations to CVD in older aged patients with PD (De Angelis et al., 2018; Singer et al., 2018). Future meta‐analyses and meta‐regressions incorporating age as a variable will improve understanding on the full impact of this risk factor.

A major limitation of the present review is the relatively small number of eligible studies and inconsistent reporting of variables such as PD severity, preventing robust conclusions. Accuracy of subgroup analysis and meta‐regression is dependent on a sufficient number of studies for analysis. There were just four European studies included in the meta‐regression by region; while this demonstrated significant accountable heterogeneity it should be interpreted with caution. The same is true for subgroup analysis by CVD outcome, with only six studies reporting risk of MI. Additionally, several included studies in the meta‐analysis did not report risk by PD severity (Chen et al., 2016; Hansen et al., 2016; Howell et al., 2001; Hujoel, Drangsholt, Spiekerman, & DeRouen, 2001; LaMonte et al., 2017; Lee et al., 2015; Lee et al., 2017; Morrison et al., 1999; Mucci et al., 2009; Wu et al., 2000) preventing full investigation of PD progression and the effect on CVD risk.

As PD can be diagnosed by a number of different classification methods and case definitions, this demonstrates a potential source of bias in determining PD prevalence within a study cohort. Recommended case definitions for PD are constantly being updated (Highfield, 2009) and there is some uncertainty on what defines periodontal health (Mariotti & Hefti, 2015). This review sought to account for this by categorising observations by PD diagnosis method (clinical/self‐report), however there are still limitations to the clinical tools used for PD diagnosis. For example, CAL may be an inappropriate measure for early clinical classification as this is a manifestation of moderate to severe PD (Tonetti, Greenwell, & Kornman, 2018). Disease symptoms can also vary by tooth type and number of sites measured, suggesting that whole mouth examination may be required for accurate PD diagnosis (Heaton, Sharma, Garcia, & Dietrich, 2018). Radiography may also not be a reliable tool for identifying PD as there is evidence it severely underestimates intra‐oral alveolar bone level (Christiaens et al., 2018). While the present review did not find differences in CVD risk by PD diagnosis method, a universal case definition for PD in research would eliminate potential for information bias and inaccurate classification of PD within clinical PD case definitions.

### 4.2 Clinical implications and future research

The findings from this systematic review reflect previous conclusions that there is a significant association between PD and CVD. We have demonstrated that the risk for CVD is high in people with PD; this risk may also be markedly increased in men and people with severe grade PD. Good practice in oral hygiene and targeting interventions in PD populations is therefore essential towards preventing CVD outcomes. Going forward, dental check‐ups could be utilised as a screening tool for both PD and CVD (Bui et al., 2019). For example, incorporation of blood pressure assessment during dental check‐ups may be a prime opportunity for CVD screening as many patients frequent dental surgeries more often than their general practitioner (Holmstrup et al., 2017). Our findings also indicate that PD precedes CVD and early diagnosis of PD along with prompt management may prevent mortality and morbidity from CVD. Part of the management of PD targets systemic risk factors, such as smoking and stress, which are also risk factors for CVD. Early interventions to target these risk factors along with PD therapy to remove causative bacterial agents might play a major role in preventing future CVD morbidity and mortality.

This review has also exposed several avenues for future research. In the short term, a universal case definition for PD is essential. Utilisation of a widespread definition will ensure continuity within dental health research, reducing bias and possible heterogeneity in meta‐analyses (Caton et al., 2018). More studies examining risk of CVD outcomes in PD, with adjustments for key confounders, are also required to permit robust meta‐analyses in the future. Recent research has demonstrated a strong association of electronic cigarette smoking and PD, with a higher risk of PD in people using e‐cigarettes compared to conventional cigarettes (Jeong et al., 2019). Smoking in addition to other risk factors such as stress should also be adjusted for in future analyses to ensure minimal confounding effects.

Patients usually have comorbidities. There is evidence showing that PD, as well as CVD, are associated with other systemic disease such as diabetes and rheumatoid arthritis. Therefore, treatment of PD may improve not only CVD but other systemic outcomes additionally (Bui et al., 2019; Falcao & Bullon, 2019; Nagpal, Yamashiro, & Izumi, 2015). If PD causes systemic disease through shared inflammatory pathways, then this could be a potential cost‐effective treatment in preventing and/or reversing CVD and other systemic diseases simultaneously.

## 5 CONCLUSION

In conclusion, the results of this systematic review and meta‐analysis demonstrate increased risk of CVD in people with PD. Males and people with severe PD have the highest risk of developing CVD indicating possible target populations for future public health interventions and screening. Further research is required to examine the cause of heterogeneity in the results and to form robust conclusions. A universal clinical case definition for PD in future cohort studies should also be adopted in order to account for possible heterogeneity.

## CONFLICT OF INTEREST

None declared.

## CLINICAL SIGNIFICANCE

### Scientific rationale for the study

Worldwide, cardiovascular disease (CVD) is a leading cause of mortality; periodontal disease (PD) is preventable yet affects up to 50% of the population. Evidence suggests the diseases share inflammatory pathways.

### Principal findings

Meta‐analysis shows consistently high risk of CVD in people with PD. Higher risk of CVD was observed in males and those with severe PD, highlighting need for effective diagnosis and management in preventing severe disease progression.

### Practical implications

Dental professionals need to manage PD effectively, or ensure prompt referral to periodontal specialists if PD does not remit, particularly for males who are at increased risk. Public health interventions are required to target PD risk factors (poor oral hygiene, smoking and stress) which also have associations with CVD.

## Supporting information


**Appendix S1.** Supporting Information.

## Data Availability

All data generated or analysed during this study are included in this published article [and its supplementary information files].

## References

[cre2336-bib-0001] Abbood, H. M. , Hinz, J. , Cherukara, G. , & Macfarlane, T. V. (2016). Validity of self‐reported periodontal disease: A systematic review and meta‐analysis. Journal of Periodontology, 87(12), 1474–1483. 10.1902/jop.2016.160196 27523519

[cre2336-bib-0002] Abnet, C. C. , Qiao, Y. L. , Dawsey, S. M. , Dong, Z. W. , Taylor, P. R. , & Mark, S. D. (2005). Tooth loss is associated with increased risk of total death and death from upper gastrointestinal cancer, heart disease, and stroke in a Chinese population‐based cohort. International Journal of Epidemiology, 34(2), 467–474. 10.1093/ije/dyh375 15659476

[cre2336-bib-0003] Aguilera, E. M. , Suvan, J. , Buti, J. , Czesnikiewicz‐Guzik, M. , Ribeiro, A. B. , Orlandi, M. , … D'Aiuto, F. (2019). Periodontitis is associated with hypertension: A systematic review and meta‐analysis. Cardiovascular Research, 116, 28–39. 10.1093/cvr/cvz201 31549149

[cre2336-bib-0004] Bahekar, A. A. , Singh, S. , Saha, S. , Molnar, J. , & Arora, R. (2007). The prevalence and incidence of coronary heart disease is significantly increased in periodontitis: A meta‐analysis. American Heart Journal, 154(5), 830–837. 10.1016/j.ahj.2007.06.037 17967586

[cre2336-bib-0005] Bale, B. F. , Doneen, A. L. , & Vigerust, D. J. (2017). High‐risk periodontal pathogens contribute to the pathogenesis of atherosclerosis. Postgraduate Medical Journal, 93(1098), 215–220. 10.1136/postgradmedj-2016-134279 27899684 PMC5520251

[cre2336-bib-0006] Baltch, A. L. , Pressman, H. L. , Schaffer, C. , Smith, R. P. , Hammer, M. C. , Shayegani, M. , & Michelsen, P. (1988). Bacteremia in patients undergoing oral procedures. Study following parenteral antimicrobial prophylaxis as recommended by the American Heart Association, 1977. Archives of Internal Medicine, 148(5), 1084–1088. 10.1001/archinte.148.5.1084 3365078

[cre2336-bib-0007] Banks, E. , Joshy, G. , Korda, R. , Stavreski, B. , Soga, K. , Egger, S. , … Lopez, A. (2019). Tobacco smoking and risk of 36 cardiovascular disease subtypes: Fatal and non‐fatal outcomes in a large prospective Australian study. BMC Medicine, 17(1), 128 10.1186/s12916-019-1351-4 31266500 PMC6607519

[cre2336-bib-0083] Batty G. D. , Jung K. J. , Mok Y. , Lee S. J. , Back J. H. , Lee S. , Jee S. H. (2018). Oral health and later coronary heart disease: Cohort study of one million people. European Journal of Preventive Cardiology, 25, (6), 598–605. 10.1177/2047487318759112. 29461088 PMC5946673

[cre2336-bib-0085] Beck J. , Garcia R ., Heiss G ., Vokonas P. S. , Offenbacher S . (1996). Periodontal Disease and Cardiovascular Disease. Journal of Periodontology, 67, (10s), 1123–1137. 10.1902/jop.1996.67.10s.1123. 8910831

[cre2336-bib-0008] Belstrom, D. , Holmstrup, P. , Damgaard, C. , Borch, T. S. , Skjodt, M. O. , Bendtzen, K. , & Nielsen, C. H. (2011). The atherogenic bacterium Porphyromonas gingivalis evades circulating phagocytes by adhering to erythrocytes. Infection and Immunity, 79(4), 1559–1565. 10.1128/iai.01036-10 21245264 PMC3067526

[cre2336-bib-0009] Beukers, N. G. , van der Heijden, G. J. , van Wijk, A. J. , & Loos, B. G. (2017). Periodontitis is an independent risk indicator for atherosclerotic cardiovascular diseases among 60 174 participants in a large dental school in The Netherlands. Journal of Epidemiol Community Health, 71(1), 37–42. 10.1136/jech-2015-206745 PMC5256268 27502782

[cre2336-bib-0010] Blicher, B. , Joshipura, K. , & Eke, P. (2005). Validation of self‐reported periodontal disease: A systematic review. Journal of Dental Research, 84(10), 881–890. 10.1177/154405910508401003 16183785

[cre2336-bib-0011] British Society of Periodontology . (2016). The Good Practitioner's Guide to Periodontology, BSP Clinical Guidelines, .Liverpool: British Society of Periodontology.

[cre2336-bib-0012] Bui, F. Q. , Almeida‐da‐Silva, C. L. C. , Huynh, B. , Trinh, A. , Liu, J. , Woodward, J. , … Ojcius, D. M. (2019). Association between periodontal pathogens and systemic disease. Biomedical Journal, 42(1), 27–35. 10.1016/j.bj.2018.12.001 30987702 PMC6468093

[cre2336-bib-0013] Caton, G. J. , Armitage, G. , Berglundh, T. , Chapple, I. L. C. , Jepsen, S. , K, S. K. , … M, S. T. (2018). A new classification scheme for periodontal and peri‐implant diseases and conditions—Introduction and key changes from the 1999 classification. Journal of Clinical Periodontology, 45(Suppl 20), S1–s8. 10.1111/jcpe.12935 29926489

[cre2336-bib-0014] Chen, D. Y. , Lin, C. H. , Chen, Y. M. , & Chen, H. H. (2016). Risk of atrial fibrillation or flutter associated with periodontitis: A nationwide, population‐based, cohort study. PLoS One, 11(10).e0165601 27798703 10.1371/journal.pone.0165601 PMC5087888

[cre2336-bib-0089] Choe H ., Kim Y. H. , Park J. W. , Kim S. Y. , Lee S , Jee S. H (2009). Tooth loss, hypertension and risk for stroke in a Korean population. Atherosclerosis, 203, (2), 550–556. 10.1016/j.atherosclerosis.2008.07.017. 19013571

[cre2336-bib-0015] Chou, S. H. , Tung, Y. C. , Lin, Y. S. , Wu, L. S. , Lin, C. P. , Liou, E. J. , … Chu, P. H. (2015). Major adverse cardiovascular events in treated periodontitis: A population‐based follow‐up study from Taiwan. PLoS One, 10(6), e0130807 10.1371/journal.pone.0130807 26114433 PMC4482590

[cre2336-bib-0016] Christiaens, V. , De Bruyn, H. , Thevissen, E. , Koole, S. , Dierens, M. , & Cosyn, J. (2018). Assessment of periodontal bone level revisited: A controlled study on the diagnostic accuracy of clinical evaluation methods and intra‐oral radiography. Clinical Oral Investigations, 22(1), 425–431. 10.1007/s00784-017-2129-8 28550521

[cre2336-bib-0017] D'Aiuto, F. , Parkar, M. , Andreou, G. , Suvan, J. , Brett, P. M. , Ready, D. , & Tonetti, M. S. (2004). Periodontitis and systemic inflammation: Control of the local infection is associated with a reduction in serum inflammatory markers. Journal of Dental Research, 83(2), 156–160. 10.1177/154405910408300214 14742655

[cre2336-bib-0018] De Angelis, F. , Basili, S. , Giovanni, F. , Dan Trifan, P. , Di Carlo, S. , & Manzon, L. (2018). Influence of the oral status on cardiovascular diseases in an older Italian population. International Journal of Immunopathology and Pharmacology, 32, 394632017751786 10.1177/0394632017751786 29363361 PMC5849242

[cre2336-bib-0019] de Boer, S. P. , Cheng, J. M. , Range, H. , Garcia‐Garcia, H. M. , Heo, J. H. , Akkerhuis, K. M. , … Kardys, I. (2014). Antibodies to periodontal pathogens are associated with coronary plaque remodeling but not with vulnerability or burden. Atherosclerosis, 237(1), 84–91. 25233105 10.1016/j.atherosclerosis.2014.08.050

[cre2336-bib-0020] DeStefano, F. , Anda, R. F. , Kahn, H. S. , Williamson, D. F. , & Russell, C. M. (1993). Dental disease and risk of coronary heart disease and mortality. BMJ, 306(6879), 688–691. 8471920 10.1136/bmj.306.6879.688 PMC1677081

[cre2336-bib-0021] Dietrich, T. , Sharma, P. , Walter, C. , Weston, P. , & Beck, J. (2013). The epidemiological evidence behind the association between periodontitis and incident atherosclerotic cardiovascular disease. Journal of Periodontology, 84(4 Suppl), S70–S84. 10.1902/jop.2013.134008 23631585

[cre2336-bib-0094] Dietrich T. , Jimenez M. , Krall Kaye E. A. , Vokonas P. S. , Garcia R. I. (2008). Age‐Dependent Associations Between Chronic Periodontitis/Edentulism and Risk of Coronary Heart Disease. Circulation, 117, (13), 1668–1674. 10.1161/circulationaha.107.711507. 18362228 PMC2582144

[cre2336-bib-0022] Falcao, A. , & Bullon, P. (2019). A review of the influence of periodontal treatment in systemic diseases. Periodontology 2000, 79(1), 117–128. 10.1111/prd.12249 30892764

[cre2336-bib-0023] Gao, S. , Li, S. , Ma, Z. , Liang, S. , Shan, T. , Zhang, M. , … Feng, X. (2016). Presence of Porphyromonas gingivalis in esophagus and its association with the clinicopathological characteristics and survival in patients with esophageal cancer. Infectious Agents and Cancer, 11, 3 10.1186/s13027-016-0049-x 26788120 PMC4717526

[cre2336-bib-0024] Gautam, D. K. , Jindal, V. , Gupta, S. C. , Tuli, A. , Kotwal, B. , & Thakur, R. (2011). Effect of cigarette smoking on the periodontal health status: A comparative, cross sectional study. Journal of Indian Society of Periodontology, 15(4), 383–387. 10.4103/0972-124x.92575 22368364 PMC3283937

[cre2336-bib-0025] Hansen, G. M. , Egeberg, A. , Holmstrup, P. , & Hansen, P. R. (2016). Relation of periodontitis to risk of cardiovascular and all‐cause mortality (from a Danish Nationwide Cohort Study). American Journal of Cardiology, 118(4), 489–493. 10.1016/j.amjcard.2016.05.036 27372888

[cre2336-bib-0026] Hawken, A. R. , Ounpuu, S. , Sliwa, K. , Zubaid, M. , Almahmeed, W. A. , … Yusuf, S. (2004). Association of psychosocial risk factors with risk of acute myocardial infarction in 11119 cases and 13648 controls from 52 countries (the INTERHEART study): Case‐control study. Lancet (London, England), 364(9438), 953–962. 10.1016/S0140-6736(04)17019-0 15364186

[cre2336-bib-0027] Heaton, B. , Gordon, N. B. , Garcia, R. I. , Rosenberg, L. , Rich, S. , Fox, M. P. , & Cozier, Y. C. (2017). A clinical validation of self‐reported periodontitis among participants in the black Women's health study. Journal of Periodontology, 88(6), 582–592. 10.1902/jop.2017.160678 28088874 PMC5556388

[cre2336-bib-0028] Heaton, B. , Sharma, P. , Garcia, R. I. , & Dietrich, T. (2018). Evaluating periodontal disease misclassification mechanisms under partial‐mouth recording protocols. Journal of Clinical Periodontology, 45(4), 422–430. 10.1111/jcpe.12874 29385644

[cre2336-bib-0091] Heitmann B.L. , Gamborg M. (2008). Remaining teeth, cardiovascular morbidity and death among adult Danes. Preventive Medicine, 47, (2), 156–160. 10.1016/j.ypmed.2008.04.007. 18534671

[cre2336-bib-0029] Heloe, L. A. (1972). Comparison of dental health data obtained from questionnaires, interviews and clinical examination. Scandinavian Journal of Dental Research, 80(6), 495–499. 4575038

[cre2336-bib-0030] Higgins, J. , Thomas, J. , Chandler, J. , Cumpston, M. , Li, T. , Page, M. , & Welch, V. (2019). Handbook for Systematic Reviews of Interventions., 2, Chichester, England: .John Wiley & Sons.

[cre2336-bib-0031] Highfield, J. (2009). Diagnosis and classification of periodontal disease. Australian Dental Journal, 54(Suppl 1), S11–S26. 10.1111/j.1834-7819.2009.01140.x 19737262

[cre2336-bib-0032] Holmlund, A. , Lampa, E. , & Lind, L. (2017). Oral health and cardiovascular disease risk in a cohort of periodontitis patients. Atherosclerosis, 262, 101–106. 28531825 10.1016/j.atherosclerosis.2017.05.009

[cre2336-bib-0033] Holmstrup, P. , Damgaard, C. , Olsen, I. , Klinge, B. , Flyvbjerg, A. , Nielsen, C. H. , & Hansen, P. R. (2017). Comorbidity of periodontal disease: Two sides of the same coin? An introduction for the clinician. Journal of Oral Microbiology, 9(1), 1332710 10.1080/20002297.2017.1332710 28748036 PMC5508374

[cre2336-bib-0034] Howell, T. H. , Ridker, P. M. , Ajani, U. A. , Hennekens, C. H. , & Christen, W. G. (2001). Periodontal disease and risk of subsequent cardiovascular disease in U.S. male physicians. Journal of the American College of Cardiology, 37(2), 445–450. 10.1016/s0735-1097(00)01130-x 11216961

[cre2336-bib-0084] Hujoel P.P. , Drangsholt M. , Spiekerman C. , DeRouen T.A. (2001). Examining the link between coronary heart disease and the elimination of chronic dental infections. The Journal of the American Dental Association, 132, (7), 883–889. 10.14219/jada.archive.2001.0300. 11480641

[cre2336-bib-0036] Hung, H. C. , Joshipura, K. J. , Colditz, G. , Manson, J. E. , Rimm, E. B. , Speizer, F. E. , & Willett, W. C. (2004). The association between tooth loss and coronary heart disease in men and women. Journal of Public Health Dentistry, 64(4), 209–215. 15562943 10.1111/j.1752-7325.2004.tb02755.x

[cre2336-bib-0037] Hung, H. C. , Willett, W. , Merchant, A. , Rosner, B. A. , Ascherio, A. , & Joshipura, K. J. (2003). Oral health and peripheral arterial disease. Circulation, 107(8), 1152–1157. 10.1161/01.cir.0000051456.68470.c8 12615794

[cre2336-bib-0038] Fenol, A. , Jebi, S. , Krishnan, S. , Perayil, J. , Vyloppillil, R. , Bhaskar A., XXX , & Mohandas, Ashitha . (2017). Association of stress, salivary cortisol level, and periodontitis among the inmates of a central prison in Kerala. Dental Research Journal (Isfahan), 14(4), 288–292. 10.4103/1735-3327.211625 PMC5553258 28928784

[cre2336-bib-0039] Jeong, W. , Choi, D. W. , Kim, Y. K. , Lee, H. J. , Lee, S. A. , Park, E. C. , & Jang, S. I. (2019). Associations of electronic and conventional cigarette use with periodontal disease in South Korean adults. Journal of Periodontology, 91, 55–64. 10.1002/jper.19-0060 31355936

[cre2336-bib-0088] Jimenez M. , Krall E. A. , Garcia R. I. , Vokonas P. S. , Dietrich T. (2009). Periodontitis and incidence of cerebrovascular disease in men. Annals of Neurology, 66, (4), 505–512. 10.1002/ana.21742. 19847898 PMC2783821

[cre2336-bib-0087] Joshy G. , Arora M. , Korda R. J , Chalmers J. , Banks E. (2016). Is poor oral health a risk marker for incident cardiovascular disease hospitalisation and all‐cause mortality? Findings from 172 630 participants from the prospective 45 and Up Study. BMJ Open, 6, (8), e012386 10.1136/bmjopen-2016-012386. PMC5013478 27577588

[cre2336-bib-0040] Joshipura, K. J. , Douglass, C. W. , Garcia, R. I. , & Valachovic, R. (1996a). Validity of a self‐reported periodontal disease measure. Journal of Public Health Dentistry, 56(4), 205–212. 10.1111/j.1752-7325.1996.tb02437.x 8906704 PMC5712839

[cre2336-bib-0041] Joshipura, K. J. , Hung, H. C. , Rimm, E. B. , Willett, W. C. , & Ascherio, A. (2003). Periodontal disease, tooth loss, and incidence of ischemic stroke. Stroke, 34(1), 47–52. 10.1161/01.str.0000052974.79428.0c 12511749

[cre2336-bib-0042] Joshipura, K. J. , Rimm, E. B. , Douglass, C. W. , Trichopoulos, D. , Ascherio, A. , & Willett, W. C. (1996b). Poor oral health and coronary heart disease. Journal of Dental Research, 75(9), 1631–1636. 8952614 10.1177/00220345960750090301

[cre2336-bib-0043] Kassebaum, N. J. , Bernabe, E. , Dahiya, M. , Bhandari, B. , Murray, C. J. , & Marcenes, W. (2014). Global burden of severe periodontitis in 1990‐2010: A systematic review and meta‐regression. Journal of Dental Research, 93(11), 1045–1053. 10.1177/0022034514552491 25261053 PMC4293771

[cre2336-bib-0044] Lafon, A. , Pereira, B. , Dufour, T. , Rigouby, V. , Giroud, M. , Bejot, Y. , & Tubert‐Jeannin, S. (2014). Periodontal disease and stroke: A meta‐analysis of cohort studies. European Journal of Neurology, 21(9), 1155–1157. 10.1111/ene.12415 24712659

[cre2336-bib-0045] LaMonte, M. J. , Genco, R. J. , Hovey, K. M. , Wallace, R. B. , Freudenheim, J. L. , Michaud, D. S. , … Wactawski‐Wende, J. (2017). History of periodontitis diagnosis and edentulism as predictors of cardiovascular disease, stroke, and mortality in postmenopausal women. Journal of the American Heart Association, 6(4), e004518 10.1161/jaha.116.004518 28356279 PMC5532989

[cre2336-bib-0046] Lee, J. H. , Oh, J. Y. , Youk, T. M. , Jeong, S. N. , Kim, Y. T. , & Choi, S. H. (2017). Association between periodontal disease and non‐communicable diseases: A 12‐year longitudinal health‐examinee cohort study in South Korea. Medicine (Baltimore), 96(26), .e7398 28658175 10.1097/MD.0000000000007398 PMC5500097

[cre2336-bib-0047] Lee, Y. L. , Hu, H. Y. , Chou, P. , & Chu, D. (2015). Dental prophylaxis decreases the risk of acute myocardial infarction: A nationwide population‐based study in Taiwan. Clinical Interventions in Aging, 10, 175–182. 25609934 10.2147/CIA.S67854 PMC4293300

[cre2336-bib-0048] Lee, Y. L. , Hu, H. Y. , Huang, N. , Hwang, D. K. , Chou, P. , & Chu, D. (2013). Dental prophylaxis and periodontal treatment are protective factors to ischemic stroke. Stroke, 44(4), 1026–1030. 23422085 10.1161/STROKEAHA.111.000076

[cre2336-bib-0049] Liao, F. , Li, Z. , Wang, Y. , Shi, B. , Gong, Z. , & Cheng, X. (2009). Porphyromonas gingivalis may play an important role in the pathogenesis of periodontitis‐associated rheumatoid arthritis. Medical Hypotheses, 72(6), 732–735. 10.1016/j.mehy.2008.12.040 19246161

[cre2336-bib-0050] Liccardo, D. , Cannavo, A. , Spagnuolo, G. , Ferrara, N. , Cittadini, A. , Rengo, C. , & Rengo, G. (2019). Periodontal disease: A risk factor for diabetes and cardiovascular disease. International Journal of Molecular Sciences, 20(6), 1414 10.3390/ijms20061414 PMC6470716 30897827

[cre2336-bib-0051] Lin, H. W. , Chen, C. M. , Yeh, Y. C. , Chen, Y. Y. , Guo, R. Y. , Lin, Y. P. , & Li, Y. C. (2019). Dental treatment procedures for periodontal disease and the subsequent risk of ischaemic stroke: A retrospective population‐based cohort study. Journal of Clinical Periodontology, 46(6), 642–649. 30989681 10.1111/jcpe.13113

[cre2336-bib-0052] Linden, G. J. , McClean, K. , Young, I. , Evans, A. , & Kee, F. (2008). Persistently raised C‐reactive protein levels are associated with advanced periodontal disease. Journal of Clinical Periodontology, 35(9), 741–747. 10.1111/j.1600-051X.2008.01288.x 18647204

[cre2336-bib-0053] Mahase, E. (2019). Cancer overtakes CVD to become leading cause of death in high income countries. BMJ, 366, l5368 10.1136/bmj.l5368 31481521

[cre2336-bib-0054] Marchetti, E. , Monaco, A. , Procaccini, L. , Mummolo, S. , Gatto, R. , Tetè, S. , … Marzo, G. (2012). Periodontal disease: The influence of metabolic syndrome. Nutrition and Metabolism (Lond), 9(1), 88 10.1186/1743-7075-9-88 PMC3499456 23009606

[cre2336-bib-0055] Mariotti, A. , & Hefti, A. F. (2015). Defining periodontal health. BMC Oral Health, 15(Suppl 1), S6 10.1186/1472-6831-15-s1-s6 26390888 PMC4580771

[cre2336-bib-0056] Mark Bartold, P. , & Mariotti, A. (2017). The future of periodontal‐systemic associations: Raising the standards. Current Oral Health Reports, 4(3), 258–262. 10.1007/s40496-017-0150-2 28944159 PMC5587612

[cre2336-bib-0057] Martin‐Cabezas, R. , Seelam, N. , Petit, C. , Agossa, K. , Gaertner, S. , Tenenbaum, H. , … Huck, O. (2016). Association between periodontitis and arterial hypertension: A systematic review and meta‐analysis. American Heart Journal, 180, 98–112. 10.1016/j.ahj.2016.07.018 27659888

[cre2336-bib-0058] Morrison, H. I. , Ellison, L. F. , & Taylor, G. W. (1999). Periodontal disease and risk of fatal coronary heart and cerebrovascular diseases. Journal of Cardiovascular Risk, 6(1), 7–11. 10.1177/204748739900600102 10197286

[cre2336-bib-0059] Mougeot, J. C. , Stevens, C. B. , Paster, B. J. , Brennan, M. T. , Lockhart, P. B. , & Mougeot, F. K. (2017). Porphyromonas gingivalis is the most abundant species detected in coronary and femoral arteries. Journal of Oral Microbiology, 9(1), 1281562 10.1080/20002297.2017.1281562 28326156 PMC5328378

[cre2336-bib-0060] Mucci, L. A. , Hsieh, C. C. , Williams, P. L. , Arora, M. , Adami, H. O. , de Faire, U. , … Pedersen, N. L. (2009). Do genetic factors explain the association between poor oral health and cardiovascular disease? A prospective study among Swedish twins. American Journal of Epidemiology, 170(5), 615–621. 10.1093/aje/kwp177 19648170 PMC2732988

[cre2336-bib-0061] Nagpal, R. , Yamashiro, Y. , & Izumi, Y. (2015). The two‐way association of periodontal infection with systemic disorders: An overview. Mediators of Inflammation, 2015, 793898 10.1155/2015/793898 26339142 PMC4539125

[cre2336-bib-0062] Nazir, M. A. (2017). Prevalence of periodontal disease, its association with systemic diseases and prevention. International Journal of Health Sciences (Qassim), 11(2), 72–80 Retrieved from https://www.ncbi.nlm.nih.gov/pmc/articles/PMC5426403/pdf/IJHS-11-72.pdf PMC5426403 28539867

[cre2336-bib-0090] Noguchi S. , Toyokawa S. , Miyoshi Y. , Suyama Y. , Inoue K. , Kobayashi Y. (2014). Five‐year follow‐up study of the association between periodontal disease and myocardial infarction among Japanese male workers: MY Health Up Study. Journal of Public Health, fdu076 10.1093/pubmed/fdu076. 25293424

[cre2336-bib-0063] Pitiphat, W. , Garcia, R. I. , Douglass, C. W. , & Joshipura, K. J. (2002). Validation of self‐reported oral health measures. Journal of Public Health Dentistry, 62(2), 122–128. 10.1111/j.1752-7325.2002.tb03432.x 11989207

[cre2336-bib-0064] Rivas‐Tumanyan, S. , Spiegelman, D. , Curhan, G. C. , Forman, J. P. , & Joshipura, K. J. (2012). Periodontal disease and incidence of hypertension in the health professionals follow‐up study. American Journal of Hypertension, 25(7), 770–776. 22476024 10.1038/ajh.2012.32 PMC3508690

[cre2336-bib-0065] Roca‐Millan, E. , Gonzalez‐Navarro, B. , Sabater‐Recolons, M. M. , Mari‐Roig, A. , Jane‐Salas, E. , & Lopez‐Lopez, J. (2018). Periodontal treatment on patients with cardiovascular disease: Systematic review and meta‐analysis. Medicina Oral, Patologia Oral, Cirugia Bucal, 23(6), e681–e690. 10.4317/medoral.22725 PMC6261003 30341272

[cre2336-bib-0066] Romandini, M. , Laforí, A. , Romandini, P. , Baima, G. , & Cordaro, M. (2018). Periodontitis and platelet count: A new potential link with cardiovascular and other systemic inflammatory diseases. Journal of Clinical Periodontology, 45(11), 1299–1310. 10.1111/jcpe.13004 30133784

[cre2336-bib-0067] Sanderson, S. , Tatt, I. D. , & Higgins, J. P. (2007). Tools for assessing quality and susceptibility to bias in observational studies in epidemiology: A systematic review and annotated bibliography. International Journal of Epidemiology, 36(3), 666–676. 10.1093/ije/dym018 17470488

[cre2336-bib-0068] Sayehmiri, F. , Sayehmiri, K. , Asadollahi, K. , Soroush, S. , Bogdanovic, L. , Jalilian, F. A. , … Taherikalani, M. (2015). The prevalence rate of Porphyromonas gingivalis and its association with cancer: A systematic review and meta‐analysis. International Journal of Immunopathology and Pharmacology, 28, 160–167 England: (c) The Author(s) 2015. 26002887 10.1177/0394632015586144

[cre2336-bib-0069] Scannapieco, F. A. , Bush, R. B. , & Paju, S. (2003). Associations between periodontal disease and risk for atherosclerosis, cardiovascular disease, and stroke. A systematic review. Annals of Periodontology, 8(1), 38–53. 10.1902/annals.2003.8.1.38 14971247

[cre2336-bib-0086] Sen S. , Giamberardino L. D. , Moss K. , Morelli T. , Rosamond W. D. , Gottesman R. F. , Beck J. , Offenbacher S. (2018). Periodontal Disease, Regular Dental Care Use, and Incident Ischemic Stroke. Stroke, 49, (2), 355–362. 10.1161/strokeaha.117.018990. 29335336 PMC5780242

[cre2336-bib-0070] Shor, E. , Roelfs, D. , & Vang, Z. M. (2017). The "Hispanic mortality paradox" revisited: Meta‐analysis and meta‐regression of life‐course differentials in Latin American and Caribbean immigrants' mortality. Social Science and Medicine, 186, 20–33. 10.1016/j.socscimed.2017.05.049 28577458

[cre2336-bib-0071] Sidhu, R. K. (2016). Association between acute myocardial infarction and periodontitis: A review of the literature. Journal of the International Academy of Periodontology, 18(1), 23–33. 26764968

[cre2336-bib-0072] Singer, R. H. , Stoutenberg, M. , Feaster, D. J. , Cai, J. , Hlaing, W. M. , Metsch, L. R. , … Schneiderman, N. (2018). The association of periodontal disease and cardiovascular disease risk: Results from the Hispanic Community Health Study/Study of Latinos. Journal of Periodontology, 89(7), 840–857. 10.1002/jper.17-0549 29542123 PMC6105526

[cre2336-bib-0073] Slots, J. (1998). Casual or causal relationship between periodontal infection and non‐oral disease? Journal of Dental Research, 77(10), 1764–1765. 10.1177/00220345980770100101 9786630

[cre2336-bib-0074] Socransky, S. S. , Haffajee, A. D. , Cugini, M. A. , Smith, C. , & Kent, R. L., Jr. (1998). Microbial complexes in subgingival plaque. Journal of Clinical Periodontology, 25(2), 134–144. 10.1111/j.1600-051x.1998.tb02419.x 9495612

[cre2336-bib-0075] Sterne, J. A. , Hernan, M. A. , Reeves, B. C. , Savovic, J. , Berkman, N. D. , Viswanathan, M. , … Higgins, J. P. (2016). ROBINS‐I: A tool for assessing risk of bias in non‐randomised studies of interventions. BMJ, 355, i4919 10.1136/bmj.i4919 27733354 PMC5062054

[cre2336-bib-0076] Sun, W. , Wu, Y. , Gao, M. , Tian, Y. , Qi, P. , Shen, Y. , … Liu, X. (2019). C‐reactive protein promotes inflammation through TLR4/NF‐kappaB/TGF‐beta pathway in HL‐1 cells. Bioscience Reports, 39(8), BSR20190888 10.1042/bsr20190888 31391207 PMC6712437

[cre2336-bib-0077] Tonetti, M. S. , Greenwell, H. , & Kornman, K. S. (2018). Staging and grading of periodontitis: Framework and proposal of a new classification and case definition. Journal of Periodontology, 89(Suppl 1), S159–s172. 10.1002/jper.18-0006 29926952

[cre2336-bib-0093] Tu Y.‐K. , Galobardes B. , Smith G. D. , McCarron P. , Jeffreys M. , Gilthorpe M. S (2007). Associations between tooth loss and mortality patterns in the Glasgow Alumni Cohort. Heart, 93, (9), 1098–1103. 10.1136/hrt.2006.097410. 17164486 PMC1955024

[cre2336-bib-0078] Viechtbauer, W. (2010). Conducting meta‐analyses in R with the metafor package. Journal of Statistical Software, 36(3), 1–48 Retrieved from http://www.jstatsoft.org/v36/i03/

[cre2336-bib-0079] Wu, T. , Trevisan, M. , Genco, R. J. , Dorn, J. P. , Falkner, K. L. , & Sempos, C. T. (2000). Periodontal disease and risk of cerebrovascular disease: The first national health and nutrition examination survey and its follow‐up study. Archives of Internal Medicine, 160(18), 2749–2755. 11025784 10.1001/archinte.160.18.2749

[cre2336-bib-0080] Xu, F. , & Lu, B. (2011). Prospective association of periodontal disease with cardiovascular and all‐cause mortality: NHANES III follow‐up study. Atherosclerosis, 218, 536–542. 21831372 10.1016/j.atherosclerosis.2011.07.091

[cre2336-bib-0081] Xu, S. , Song, M. , Xiong, Y. , Liu, X. , He, Y. , & Qin, Z. (2017). The association between periodontal disease and the risk of myocardial infarction: A pooled analysis of observational studies. BMC Cardiovascular Disorders, 17(1), 50 10.1186/s12872-017-0480-y 28143450 PMC5286862

[cre2336-bib-0082] Yang, S. , Zhao, L. S. , Cai, C. , Shi, Q. , Wen, N. , & Xu, J. (2018). Association between periodontitis and peripheral artery disease: A systematic review and meta‐analysis. BMC Cardiovascular Disorders, 18(1), 141 10.1186/s12872-018-0879-0 29980169 PMC6035462

[cre2336-bib-0092] Yu Y‐H , Chasman D I. , Buring JE. , Rose L , Ridker PM (2015). Cardiovascular risks associated with incident and prevalent periodontal disease. Journal of Clinical Periodontology, 42, (1), 21–28. 10.1111/jcpe.12335. 25385537 PMC4300240

