# Supplementary material for: Risk of incident cardiovascular disease in people with periodontal disease: A systematic review and meta‐analysis
Source: Clin Exp Dent Res. 2020 Oct 30;7(1):109–22. doi: 10.1002/cre2.336 (PMC7853902; doi:10.1002/cre2.336)
Supplement: Supplementary file 1 — Appendix S1. Supporting Information. [file CRE2-7-109-s001.zip › CRE2_336_cre2.20200202-File008.pdf]

Title: Risk of incident cardiovascular disease in people with periodontal disease: a systematic review and meta-analysis.

Authors: Harriet Larvin[1], Jing Kang[2], Vishal. R. Aggarwal[1], Sue Pavitt[1], Jianhua Wu[1,3]

1. School of Dentistry, University of Leeds, Leeds, UK.
2. Oral Biology, School of Dentistry, University of Leeds, Leeds, UK
3. Leeds Institute for Data Analytics, University of Leeds, Leeds, UK

Correspondence to:

Jianhua Wu

Worsley Building, Level 6

Clarendon Way, University of Leeds

Leeds, UK, LS2 9LU

j.h.wu@leeds.ac.uk

Tel: +44 113 343 3431

Supplementary Table 1 Example of search strategy

"Periodontal disease" AND "cardiovascular disease" AND "incidence" AND "longitudinal/randomised controlled trial design"

Search strategy for Ovid MEDLINE(r) In- Process & Other Non-Indexed Citations and Ovid MEDLINE(R): 1946 to Present.

1. periodon.tw.*
2. tooth loss.tw.
3. missing teeth.tw.
4. exp periodontal disease/
5. 1 or 2 or 3 or 4
6. atrial fibrillation.tw.
7. heart failure.tw.
*8. *cerebrovascular accident/*
9. stroke.tw.
10. angina.tw.
11. acute coronary syndrome.tw.
12. peripheral vascular disease.tw.
13. hypertension.tw.
14. exp cardiovascular disease/
15. 6 or 7 or 8 or 9 or 10 or 11 or 12 or 13 or 14
*16. *incidence/*
17. incidence.tw.
18. exp cohort analysis/
19. longitudinal.tw.
*20. *randomized controlled trial/*
21. rct.tw.
*22. randomi*ed controlled trial.tw.*
23. 16 or 17
24. 18 or 19 or 20 or 21 or 22
25. 5 and 15 and 23 and 24
