# Supplementary material for: Risk of incident cardiovascular disease in people with periodontal disease: A systematic review and meta‐analysis
Source: Clin Exp Dent Res. 2020 Oct 30;7(1):109–22. doi: 10.1002/cre2.336 (PMC7853902; doi:10.1002/cre2.336)
Supplement: Supplementary file 1 — Appendix S1. Supporting Information. [file CRE2-7-109-s001.zip › Suppl_TablesAndFig_v3.pdf]

Title: Risk of incident cardiovascular disease in people with periodontal disease: a systematic review and meta-analysis.

Authors: Harriet Larvin[1], Jing Kang[2], Vishal. R. Aggarwal[1], Sue Pavitt[1], Jianhua Wu[1,3]

1. School of Dentistry, University of Leeds, Leeds, UK.
2. Oral Biology, School of Dentistry, University of Leeds, Leeds, UK
3. Leeds Institute for Data Analytics, University of Leeds, Leeds, UK

Correspondence to:

Jianhua Wu

Worsley Building, Level 6

Clarendon Way, University of Leeds

Leeds, UK, LS2 9LU

j.h.wu@leeds.ac.uk

Tel: +44 113 343 3431

Supplementary Table 1 Example of search strategy

"Periodontal disease" AND "cardiovascular disease" AND "incidence" AND "longitudinal/randomised controlled trial design"

Search strategy for Ovid MEDLINE(r) In- Process & Other Non-Indexed Citations and Ovid MEDLINE(R): 1946 to Present.

1. periodon.tw.*
2. tooth loss.tw.
3. missing teeth.tw.
4. exp periodontal disease/
5. 1 or 2 or 3 or 4
6. atrial fibrillation.tw.
7. heart failure.tw.
*8. *cerebrovascular accident/*
9. stroke.tw.
10. angina.tw.
11. acute coronary syndrome.tw.
12. peripheral vascular disease.tw.
13. hypertension.tw.
14. exp cardiovascular disease/
15. 6 or 7 or 8 or 9 or 10 or 11 or 12 or 13 or 14
*16. *incidence/*
17. incidence.tw.
18. exp cohort analysis/
19. longitudinal.tw.
*20. *randomized controlled trial/*
21. rct.tw.
*22. randomi*ed controlled trial.tw.*
23. 16 or 17
24. 18 or 19 or 20 or 21 or 22
25. 5 and 15 and 23 and 24

Supplementary Table 2 Summary table of included studies.

Study	Region	Study design	Data Source	Population (n)	Follow Up (years)	Sex	Outcome	PD Diagnosis method	PD Severity	PD (n)	Non-PD (n)	PD Outcome (n)	Non-PD Outcome (n)	RR	RRCI	Risk of Bias*	Notes
**Abnet 2005**	China	Prospective	General population trial	29584	15	Both	Stroke	Clinical	Any	NR	NR	NR	NR	1.11	1.01-1.23	Critical	Not eligible for meta-analysis as raw data not extractable.
							CHD			NR	NR	NR	NR	1.28	1.17-1.4		
**Batty 2018**	South Korea	Prospective	Korean Cancer Prevention Study	626106	21	Male	CHD	Clinical	Mild	151413	439102	11972	29876	1.05	1.00-1.10	Serious	Converted HR to RR.
									Moderate	24311	439102	2258	29876	1.09	1.02-1.17		
									Severe	11280	439102	1176	29876	1	0.95-1.06		
**Beck 1996**	USA	Prospective	VA Normative Aging and Dental Longitudinal Study	1147	18	Male	CHD	Clinical	Severe	243	904	NR	NR	1.47	1.04-2.04	Critical	Converted OR to RR. Did not adjust for smoking.
							Stroke							2.59	1.42-4.57		
**Chen 2016**	Taiwan	Retrospective	National Health Insurance Research Database	787490	10	Both	AF	Clinical	Any	393745	393745	8138	6180	1.31	1.24-1.36	Critical	Converted HR to RR.
**Choe 2009**	South Korea	Prospective	Korean Cancer Prevention Study	679170	14	Male	Stroke	Clinical	Mild	175747	462695	7210	13463	1.10	1.10-1.11	Critical	Converted HR to RR.
									Moderate	28245	462695	1808	13463	1.29	1.20-1.29		
									Severe	12483	462695	1071	13463	1.29	1.20-1.39		
						Female			Mild	31312	146227	729	3287	1.00	0.90-1.00		
									Moderate	5946	146227	282	3287	1.10	1.00-1.30		
									Severe	4601	146227	408	3287	1.20	1.00-1.30		
**Chou 2015**	Taiwan	Retrospective	National Health Insurance Research Database	27146	9	Both	CVD	Clinical	Severe	13573	13573	1206	728	1.24	1.08-1.42	Critical	PD was treated during follow up period. Converted OR to RR. Did not adjust for smoking.
**DeStefano 1993**	USA	Prospective	NHANES 1 and epidemiological follow up	9760	16	Both	CHD	Clinical	Mild	2282	3542	170	231	1.05	0.88-1.26	Serious	Cause of death as CHD also analysed separately. Did not adjust for smoking for all included participants.
									Moderate	1786	3542	358	231	1.25	1.06-1.48		
									Severe	2150	3542	413	231	1.23	1.05-1.44		
						Male			Mild	895	1096	NR	NR	0.98	0.63-1.54		
									Moderate	922	1096	NR	NR	1.72	1.10-2.68		
									Severe	874	1096	NR	NR	1.71	0.93-3.15		
	USA	Prospective		1203	35	Male	CHD	Clinical	Mild	140	131	63	48	1.08	0.81-1.43	Critical	

Study	Region	Study design	Data Source	Population (n)	Follow Up (years)	Sex	Outcome	PD Diagnosis method	PD Severity	PD (n)	Non-PD (n)	PD Outcome (n)	Non-PD Outcome (n)	RR	RRCI	Risk of Bias*	Notes
**Dietrich 2008**			VA Normative Aging and Dental Longitudinal Study						Moderate	91	131	47	48	1.17	0.85-1.56		Raw 2 x 2 data calculated by proportion of person years. Converted HR to RR.
									Severe	78	131	26	48	0.77	0.51-1.14		
**Hansen 2016**	Denmark	Prospective	Danish National Patient Register	100694	15	Both	MI	Clinical	Any	17691	83003	NR	NR	1.16	1.04-1.30	Critical	
							Stroke			17691	83003	NR	NR	1.51	1.38-1.65		
							CVD			17691	83003	NR	NR	1.55	1.47-1.65		
**Heitmann 2008**	Denmark	Prospective	MONICA study	2932	7	Male	CVD	Clinical	Severe	147	507	49	43	1.48	0.90-2.36	Serious	Sub group analyses by CVD event and males vs. females. Converted HR to RR.
									Moderate	167	507	45	43	1.61	1.02-2.48		
									Mild	318	507	53	43	1.20	0.80-1.80		
							CHD		Severe	141	674	28	21	1.73	0.88-3.33		
									Moderate	183	674	25	21	1.21	0.63-2.29		
									Mild	303	674	26	21	1.24	0.68-2.23		
							Stroke		Severe	144	542	14	8	2.40	0.95-6.04		
									Moderate	249	542	12	8	1.34	0.52-3.38		
									Mild	382	542	10	8	1.07	0.42-2.72		
						Female	CVD		Severe	133	565	39	41	1.59	0.91-2.73		
									Moderate	233	565	36	41	1.01	0.60-1.67		
									Mild	256	565	30	41	1.25	0.76-2.02		
							CHD		Severe	153	726	14	11	0.57	0.19-1.69		
									Moderate	121	726	9	11	1.10	0.40-2.96		
									Mild	342	726	7	11	0.42	0.15-1.21		
							Stroke		Severe	152	952	14	6	5.25	1.97-13.72		
									Moderate	95	952	7	6	4.39	1.45-12.99		
									Mild	154	952	7	6	3.01	1.01-8.93		
**Holmlund 2017**	Sweden	Prospective	Patients seeking PD treatment at Gavle County Hospital	8999	34	Both	CVD	Clinical	Mild	NR	NR	NR	NR	1.18	0.95-1.43	Critical	Not eligible for meta-analysis as data not extractable (estimates taken).
									Moderate	NR	NR	NR	NR	1.35	1.10-1.64		
									Moderate/Severe	NR	NR	NR	NR	1.16	0.94-1.42		
									Severe	NR	NR	NR	NR	1.28	1.04-1.57		

Study	Region	Study design	Data Source	Population (n)	Follow Up (years)	Sex	Outcome	PD Diagnosis method	PD Severity	PD (n)	Non-PD (n)	PD Outcome (n)	Non-PD Outcome (n)	RR	RRCI	Risk of Bias*	Notes
							MI		Mild	NR	NR	NR	NR	0.91	0.68-1.22		
									Moderate	NR	NR	NR	NR	1.11	0.83-1.47		
									Moderate/Severe	NR	NR	NR	NR	1.10	0.83-1.46		
									Severe	NR	NR	NR	NR	1.11	0.83-1.47		
							Stroke		Mild	NR	NR	NR	NR	1.18	0.86-1.61		
									Moderate	NR	NR	NR	NR	1.37	1.01-1.87		
									Moderate/Severe	NR	NR	NR	NR	1.08	0.78-1.5		
									Severe	NR	NR	NR	NR	1.29	0.93-1.77		
							HF		Mild	NR	NR	NR	NR	1.40	0.92-2.14		
									Moderate	NR	NR	NR	NR	1.75	1.15-2.66		
									Moderate/Severe	NR	NR	NR	NR	1.18	0.75-1.87		
									Severe	NR	NR	NR	NR	1.71	1.12-2.62		
**Howell 2001**	USA	RCT	Physicians' Health Study I	22037	13	Male	MI	Proxy	Any	2653	19384	115	682	1.01	0.82-1.24	Critical	Fatal CVD events reported separately (using death certificate etc.)
							Stroke			2653	19384	94	537	1.01	0.81-1.27		
**Hujoel 2001**	USA	Prospective	NHANES 1 and epidemiological follow up	8032	20	Both	CHD	Clinical	Any	1859	3752	NR	NR	1.14	0.98-1.34	Serious	Mortality data reported separately.
**Hung 2004**	USA	Retrospective	The Health Professionals Follow Up Study, Nurses' Health Study	100381	12	Male	CHD	Proxy	Mild	4532	34788	254	1222	1.10	0.95-1.26	Critical	Uses two separate studies in analysis.
									Moderate	904	34788	71	1222	1.35	1.06-1.72		
									Severe	1183	34788	107	1222	1.36	1.11-1.67		
						Female			Mild	11883	38032	123	241	1.14	0.92-1.42		
									Moderate	2925	38032	43	241	1.34	0.97-1.87		
									Severe	6134	38032	137	241	1.64	1.31-2.05		
**Hung 2003**	USA	Prospective	The Health Professionals Follow-up Study	45094	12	Male	PAD	Proxy	Mild	4998	37781	65	234	1.19	0.90-1.58	Critical	Also compared with incident tooth loss and risk of PAD.
									Moderate	991	37781	19	234	1.43	0.89-2.31		
									Severe	1324	37781	24	234	1.05	0.68-1.63		
	USA	Prospective		1231	34	Male	Stroke	Clinical	Moderate	456	414	30	24	0.89	0.52-1.53	Critical	Converted HR to RR.

Study	Region	Study design	Data Source	Population (n)	Follow Up (years)	Sex	Outcome	PD Diagnosis method	PD Severity	PD (n)	Non-PD (n)	PD Outcome (n)	Non-PD Outcome (n)	RR	RRCI	Risk of Bias*	Notes
**Jimenez 2009**			VA Normative Aging and Dental Longitudinal Study						Severe	264	414	25	24	1.07	0.90-1.26		
**Joshipura 2003**	USA	Prospective	The Health Professionals Follow-up Study	41380	12	Male	Stroke	Proxy	Mild	4527	34767	72	230	1.49	1.14-1.95	Critical	Association of diet was also explored. Converted HR to RR.
									Moderate	903	34767	19	230	1.68	1.04-2.68		
									Severe	1183	34767	28	230	1.62	1.08-2.44		
**Joshipura 1996**	USA	Prospective	The Health Professionals Follow-up Study	44119	6	Male	CHD	Proxy	Mild	4815	37079	117	554	1.03	0.83-1.27	Critical	Subgroup analysis investigating how tooth loss is associated to CHD in men with PD history only.
									Moderate	950	37079	29	554	1.04	0.71-1.54		
									Severe	1275	37079	57	554	1.29	0.96-1.73		
**Joshy 2016**	Australia	Prospective	Sax Institute 45 and Up study	172630	5	Both	CHD	Proxy	Mild	30013	117464	708	1812	1.05	0.96-1.15	Serious	Converted HR to RR.
									Moderate	11423	117464	354	1812	1.20	1.06-1.35		
									Severe	8797	117464	258	1812	1.10	0.95-1.26		
							HF		Mild	30013	117464	55	67	1.50	1.04-2.18		
									Moderate	11423	117464	43	67	2.04	1.35-3.09		
									Severe	8797	117464	38	67	1.97	1.27-3.07		
							PVD		Mild	30013	117464	98	127	1.67	1.27-2.19		
									Moderate	11423	117464	53	127	1.73	1.23-2.44		
									Severe	8797	117464	65	127	2.53	1.81-3.52		
							Stroke		Mild	30013	117464	72	145	1.11	0.72-1.73		
									Moderate	11423	117464	27	145	0.90	0.59-1.40		
									Severe	8797	117464	27	145	1.20	0.90-1.62		
**LaMonte 2017**	USA	Prospective	Women's Health Initiative Observational Study	57001	12	Female	CVD	Proxy	Any	14847	42154	949	2640	1.06	0.98-1.13	Critical	Converted OR to RR.
							CHD			14847	42154	450	1238	1.08	0.97-1.19		
							Stroke			14847	42154	226	612	1.11	0.95-1.29		
**Lee 2017**	South Korea	Retrospective	Korean National Health iIsurance Database	354850	12	Both	Hypertension	Clinical	Any	154824	200026	98373	78254	1.02	1.00-1.04	Critical	Converted OR to RR.
							Stroke			154824	200026	19874	17896	0.98	0.95-1.03		
							Angina			154824	200026	33932	26259	1.19	1.14-1.23		
							MI			154824	200026	4508	3656	0.88	0.81-0.97		

Study	Region	Study design	Data Source	Population (n)	Follow Up (years)	Sex	Outcome	PD Diagnosis method	PD Severity	PD (n)	Non-PD (n)	PD Outcome (n)	Non-PD Outcome (n)	RR	RRCI	Risk of Bias*	Notes
**Lee 2015**	Taiwan	Retrospective	National Health Insurance Research Database	720343	10	Both	MI	Clinical	Any	45575	208713	534	4327	1.23	1.13-1.35	Critical	Converted HR to RR. Did not adjust for smoking.
**Lee 2013**	Taiwan	Retrospective	National Health Insurance Research Database	719436	10	Both	Stroke	Clinical	Any	45296	208674	814	7223	1.15	1.069-1.23	Critical	Converted HR to RR. Did not adjust for smoking.
**Lin 2019**	Taiwan	Retrospective	National Health Insurance Research Database	161923	10	Both	Stroke	Clinical	Moderate	87407	74516	7550	3787	1.16	1.04-1.28	Critical	Converted HR to RR. Did not adjust for smoking.
**Morrison 1999**	Canada	Retrospective	Nutrition Canada Survey	9331	23	Both	CHD	Clinical	Any	1340	1113	63	19	1.37	0.80-2.35	Serious	
							Stroke			1340	1113	33	8	1.63	0.72-3.67		
**Mucci 2009**	Sweden	Prospective	Swedish Twin Registry	15273	37	Both	CVD	Proxy	Any	1266	8654	677	534	1.29	1.00-1.38	Serious	Converted HR to RR.
							CHD			1266	8654	297	186	1.39	1.10-1.59		
							Stroke			1266	8654	71	94	1.00	0.70-1.40		
**Noguchi 2015**	Japan	Prospective	MY Health Up study	3081	5	Male	MI	Proxy	Severe	468	2619	10	7	1.96	0.71-5.39	Serious	
									Any	739	2345	9	8	2.25	0.84-5.92		
**Rivas-Tumanyan 2012**	USA	Prospective	The Health Professionals Follow-up Study	31543	20	Male	Hypertension	Proxy	Any	4641	26902	NR	NR	1.07	1.01-1.13	Critical	Converted OR to RR.
									Severe	732	27112	NR	NR	1.05	0.91-1.21		
**Sen 2018**	USA	Retrospective	Dental Atherosclerosis Risk in Communities Study	6736	15	Both	Stroke	Clinical	Mild	1036	1837	NR	NR	1.82	1.16-2.84	Serious	Converted HR to RR.
									Moderate	793	1837	NR	NR	1.98	1.25-3.10		
									Severe	890	1837	NR	NR	2.03	1.28-3.18		
**Tu 2007**	UK	Prospective	Glasgow Alumni Cohort	12631	57	Both	CVD	Clinical	Moderate	3192	7403	165	319	1.14	0.94-1.38	Serious	Converted HR to RR.
									Severe	953	7403	73	319	1.34	1.03-1.74		
							CHD		Moderate	3192	7403	118	222	1.18	0.94-1.49		
									Severe	953	7403	44	222	1.19	0.84-1.67		
							Stroke		Moderate	3192	7403	25	64	0.88	0.55-1.41		
									Severe	953	7403	20	64	1.64	0.96-2.78		
**Wu 2000**	USA	Prospective		9962	22	Both	Stroke	Clinical	Mild	2346	3634	121	158	1.02	0.70-1.48	Serious	

Study	Region	Study design	Data Source	Population (n)	Follow Up (years)	Sex	Outcome	PD Diagnosis method	PD Severity	PD (n)	Non-PD (n)	PD Outcome (n)	Non-PD Outcome (n)	RR	RRCI	Risk of Bias*	Notes
			NHANES 1 and epidemiological follow up.						Moderate	1800	3634	194	158	1.66	1.15-2.39		Fatal events examined separately.
									Severe	2182	3634	330	158	1.23	0.91-1.66		
**Yu 2015**	USA	Prospective	Women's Health Initiative Observational Study	39863	16	Female	MI	Proxy	Any	7185	29787	NR	NR	1.34	1.05-1.70	Critical	Converted HR to RR.
							Stroke			7185	29787	NR	NR	1.05	0.82-1.34		

Note: atrial fibrillation (AF), coronary heart disease (CHD), confidence interval (CI), cardiovascular disease (CVD), heart failure (HF), hazard ratio (HR), international classification of diseases (ICD), myocardial infarction (MI), number (n), national health and nutrition examination survey (NHANES), not reported (NR), odds ratio (OR), peripheral artery disease (PAD), periodontal disease (PD), peripheral vascular disease (PVD), relative risk (RR), veterans affairs (VA).

Risk of bias was defined according to ROBINS-I assessment (*)

Supplementary Table 3 Results of the assessment of risk of bias in included observational studies (ROBINS)

Study ID	Bias caused by confounding	Bias caused by selection	Bias caused by classification of exposure	Bias caused by deviations from intended exposure	Attrition bias caused by missing data	Detection bias caused by measurement of outcomes	Reporting bias caused by selection of the reported results	Overall judgement
Abnet 2005	MODERATE	MODERATE	CRITICAL	CRITICAL	CRITICAL	SERIOUS	SERIOUS	CRITICAL
Batty 2018	MODERATE	CRITICAL	MODERATE	SERIOUS	SERIOUS	SERIOUS	SERIOUS	SERIOUS
Beck 1996	CRITICAL	CRITICAL	MODERATE	MODERATE	SERIOUS	MODERATE	SERIOUS	CRITICAL
Chen 2016	CRITICAL	SERIOUS	MODERATE	MODERATE	SERIOUS	MODERATE	SERIOUS	CRITICAL
Choe 2009	MODERATE	CRITICAL	MODERATE	SERIOUS	SERIOUS	MODERATE	SERIOUS	CRITICAL
Chou 2015a	CRITICAL	MODERATE	MODERATE	MODERATE	SERIOUS	MODERATE	SERIOUS	CRITICAL
DeStefano 1993	SERIOUS	MODERATE	MODERATE	MODERATE	SERIOUS	MODERATE	SERIOUS	SERIOUS
Dietrich 2008	MODERATE	CRITICAL	MODERATE	MODERATE	SERIOUS	MODERATE	SERIOUS	CRITICAL
Hansen 2016	MODERATE	MODERATE	MODERATE	MODERATE	CRITICAL	MODERATE	SERIOUS	CRITICAL
Heitmann 2008	MODERATE	MODERATE	MODERATE	MODERATE	SERIOUS	SERIOUS	SERIOUS	SERIOUS
Holmlund 2017	MODERATE	MODERATE	MODERATE	MODERATE	CRITICAL	MODERATE	SERIOUS	CRITICAL
Howell 2001	MODERATE	CRITICAL	SERIOUS	SERIOUS	SERIOUS	SERIOUS	SERIOUS	CRITICAL
Hujoel 2001	MODERATE	MODERATE	MODERATE	MODERATE	SERIOUS	MODERATE	SERIOUS	SERIOUS
Hung 2003	MODERATE	CRITICAL	SERIOUS	SERIOUS	SERIOUS	SERIOUS	SERIOUS	CRITICAL
Hung 2004	MODERATE	CRITICAL	SERIOUS	SERIOUS	SERIOUS	SERIOUS	SERIOUS	CRITICAL
Jimenez 2009	MODERATE	CRITICAL	MODERATE	MODERATE	SERIOUS	MODERATE	SERIOUS	CRITICAL
Joshipura 1996	MODERATE	CRITICAL	SERIOUS	SERIOUS	SERIOUS	SERIOUS	SERIOUS	CRITICAL
Joshipura 2003	MODERATE	CRITICAL	SERIOUS	SERIOUS	SERIOUS	SERIOUS	SERIOUS	CRITICAL
Joshy 2016	MODERATE	MODERATE	SERIOUS	SERIOUS	SERIOUS	SERIOUS	SERIOUS	SERIOUS
LaMonte	MODERATE	CRITICAL	CRITICAL	SERIOUS	CRITICAL	SERIOUS	SERIOUS	CRITICAL
Lee 2013	CRITICAL	MODERATE	MODERATE	MODERATE	SERIOUS	MODERATE	SERIOUS	CRITICAL
Lee 2015a	CRITICAL	MODERATE	MODERATE	MODERATE	SERIOUS	SERIOUS	SERIOUS	CRITICAL
Lee 2017	CRITICAL	MODERATE	MODERATE	MODERATE	SERIOUS	MODERATE	SERIOUS	CRITICAL
Lin 2019	CRITICAL	MODERATE	MODERATE	MODERATE	SERIOUS	MODERATE	SERIOUS	CRITICAL
Morrison 1999	MODERATE	MODERATE	MODERATE	MODERATE	SERIOUS	MODERATE	SERIOUS	SERIOUS
Mucci 2009	MODERATE	SERIOUS	SERIOUS	SERIOUS	SERIOUS	MODERATE	SERIOUS	SERIOUS
Noguchi 2015	MODERATE	SERIOUS	SERIOUS	SERIOUS	SERIOUS	SERIOUS	SERIOUS	SERIOUS
Rivas-Tumanyan 2012	MODERATE	CRITICAL	MODERATE	MODERATE	SERIOUS	MODERATE	SERIOUS	CRITICAL
Sen 2018	MODERATE	MODERATE	MODERATE	MODERATE	SERIOUS	MODERATE	SERIOUS	SERIOUS
Tu 2007	MODERATE	MODERATE	SERIOUS	MODERATE	SERIOUS	MODERATE	SERIOUS	SERIOUS
Wu 2000	MODERATE	MODERATE	MODERATE	MODERATE	SERIOUS	MODERATE	SERIOUS	SERIOUS
Yu 2015	MODERATE	CRITICAL	SERIOUS	SERIOUS	SERIOUS	SERIOUS	SERIOUS	CRITICAL

Supplementary Figure 1 A funnel plot demonstrating publication bias.

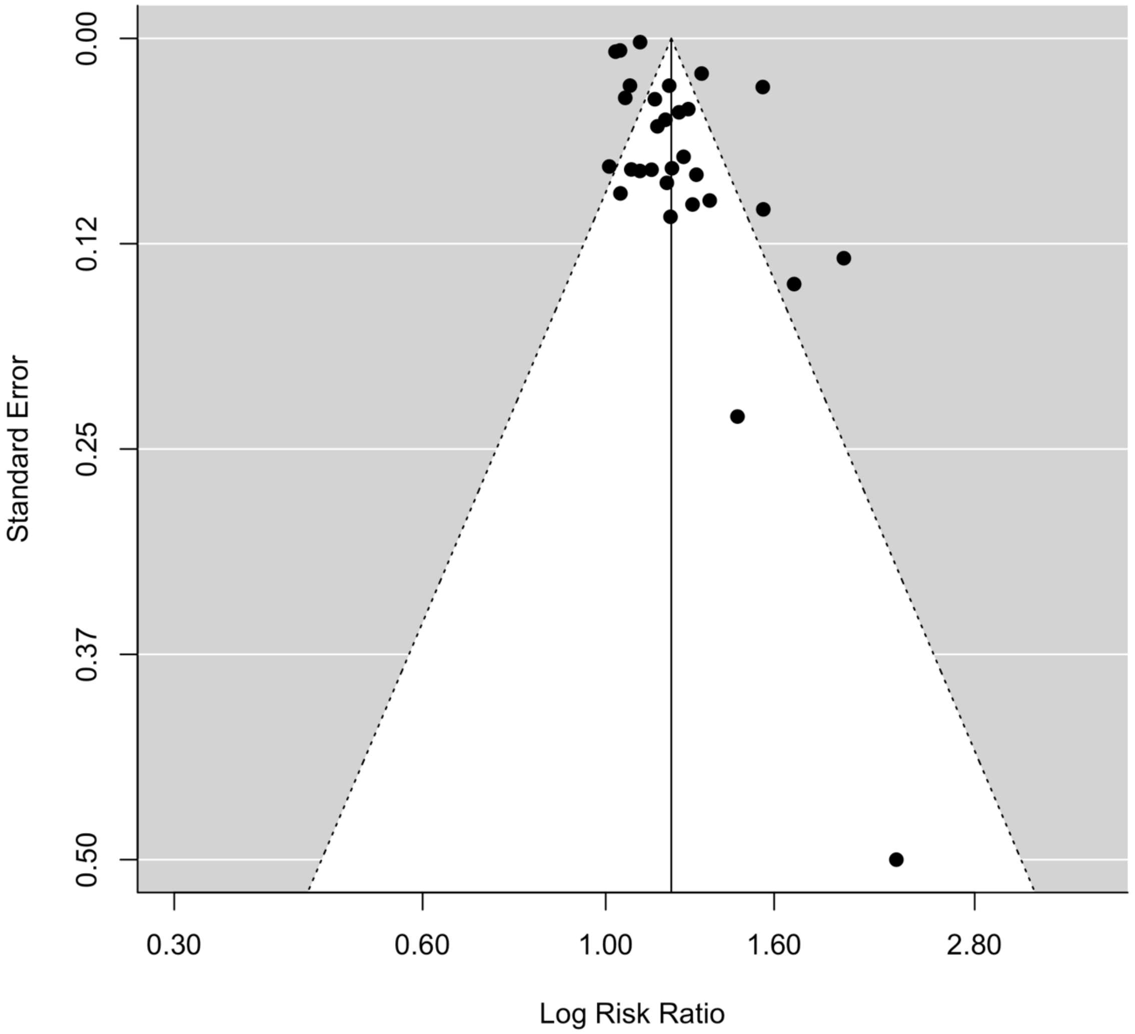

Supplementary Figure 2 Forest plot illustrating results from random effect meta-analysis for the incident risk of stroke in people with periodontal disease (PD) and by PD diagnosis method.

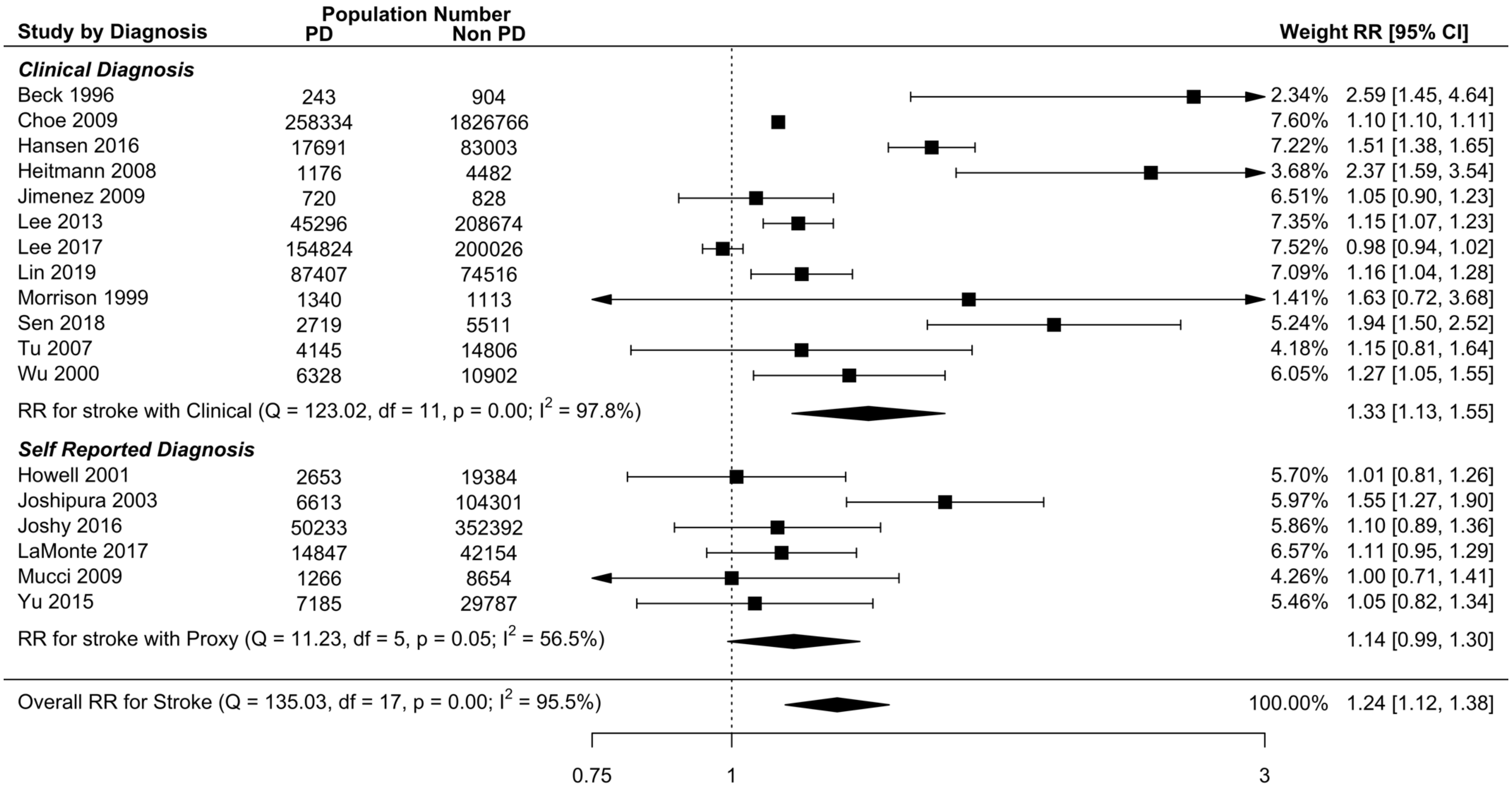

Supplementary Figure 3 Forest plot illustrating results from random effect meta-analysis for the incident risk of coronary heart disease (CHD) in people with PD and by PD diagnosis method.

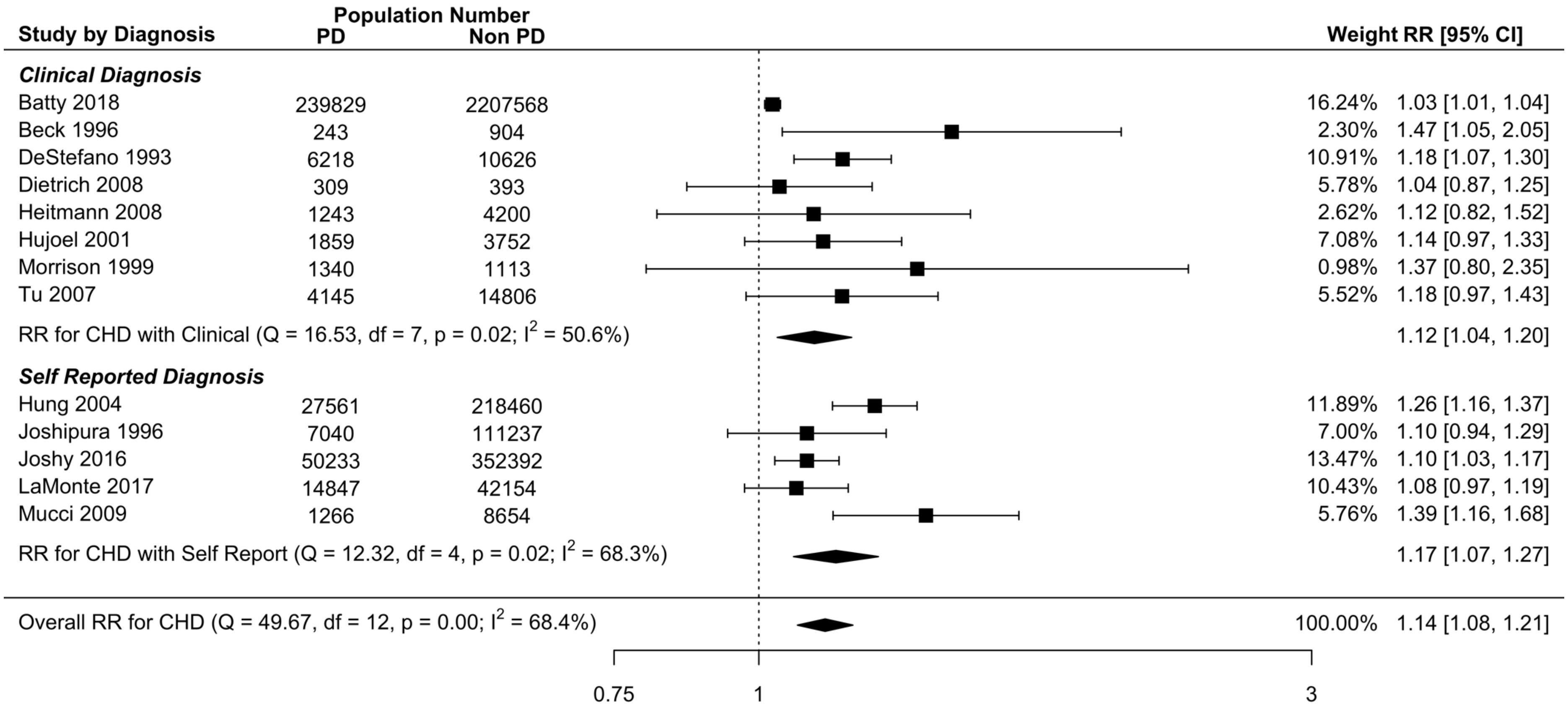

Supplementary Figure 4 Forest plot illustrating results from random effect meta-analysis for the incident risk of myocardial infarction (MI) in people with PD and by PD diagnosis method.

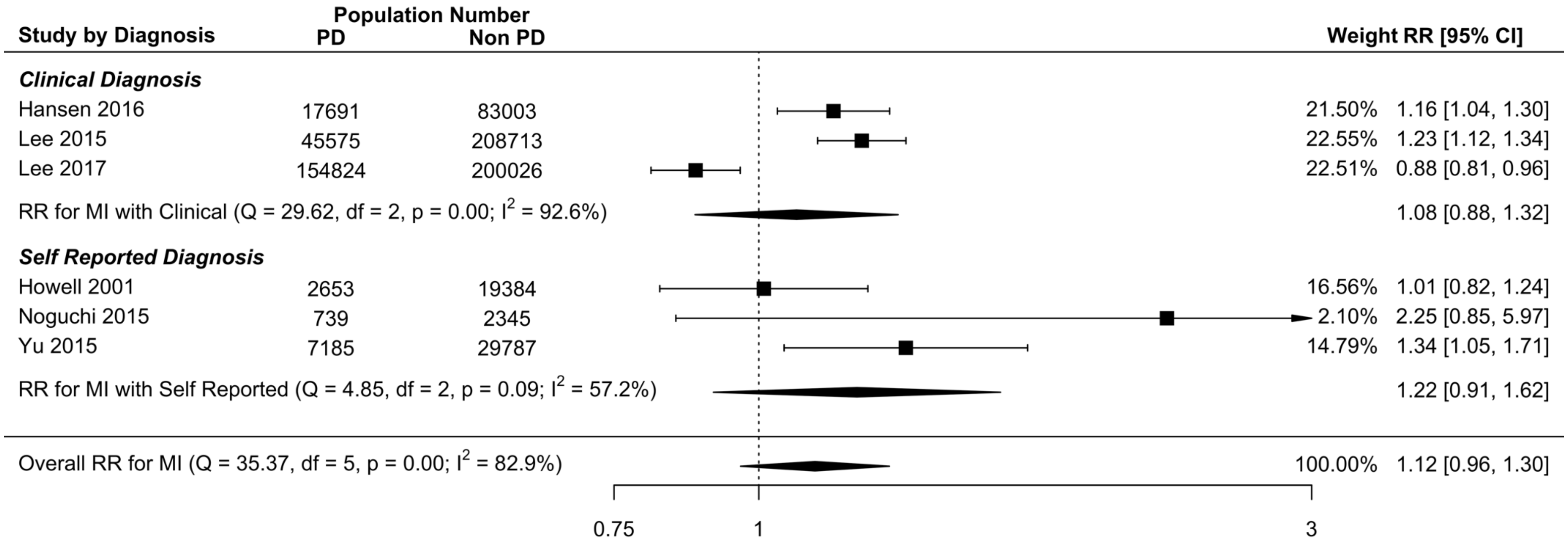

Supplementary Figure 5 Forest plot illustrating results from sensitivity analysis for the incident risk of all cardiovascular disease (CVD) in people with PD in only studies that adjusted for smoking.

Study by Diagnosis	Population Number PD	Population Number Non PD	Weight	RR [95% CI]
Clinical Diagnosis				
Batty 2018	239829	2207568	5.29%	1.03 [1.01, 1.04]
Beck 1996	486	1808	2.28%	1.69 [1.26, 2.26]
Chen 2016	393745	393745	5.17%	1.31 [1.25, 1.36]
Choe 2009	258334	1826766	5.31%	1.10 [1.10, 1.11]
Dietrich 2008	309	393	3.47%	1.04 [0.87, 1.25]
Hansen 2016	17691	83003	5.05%	1.55 [1.46, 1.64]
Heitmann 2008	1254	3216	3.36%	1.34 [1.10, 1.62]
Hujoel 2001	1859	3752	3.84%	1.14 [0.97, 1.33]
Jimenez 2009	720	828	3.80%	1.05 [0.90, 1.23]
Lee 2017	619296	800104	5.29%	1.04 [1.03, 1.06]
Lin 2019	87407	74516	4.53%	1.16 [1.04, 1.28]
Morrison 1999	2680	2226	1.28%	1.44 [0.92, 2.26]
Sen 2018	2719	5511	2.57%	1.94 [1.50, 2.52]
Tu 2007	4145	14806	3.87%	1.20 [1.03, 1.40]
Wu 2000	6328	10902	3.30%	1.27 [1.05, 1.55]
RR for CVD with Clinical (Q = 362.54, df = 14, p = 0.00; I^2 = 99.2%)				1.23 [1.13, 1.35]
Self Reported Diagnosis				
Howell 2001	5306	38768	3.90%	1.01 [0.87, 1.18]
Hung 2003	7313	113343	3.12%	1.20 [0.97, 1.48]
Hung 2004	27561	218460	4.78%	1.26 [1.16, 1.37]
Joshipura 1996	7040	111237	3.82%	1.10 [0.94, 1.29]
Joshipura 2003	6613	104301	3.22%	1.55 [1.27, 1.90]
Joshy 2016	200932	1409568	5.06%	1.19 [1.13, 1.26]
LaMonte 2017	14847	42154	4.92%	1.06 [0.98, 1.13]
Mucci 2009	1266	8654	3.76%	1.29 [1.10, 1.51]
Noguchi 2015	739	2345	0.33%	2.25 [0.85, 5.97]
Rivas-Tumanyan 2012	4641	26902	5.06%	1.07 [1.01, 1.13]
Yu 2015	14370	59574	3.63%	1.19 [1.00, 1.41]
RR for CVD with Self Reported (Q = 33.32, df = 10, p = 0.00; I^2 = 72.7%)				1.17 [1.09, 1.25]
Overall RR for CVD (Q = 403.68, df = 25, p = 0.00; I^2 = 98.1%)			100.00%	1.20 [1.14, 1.28]

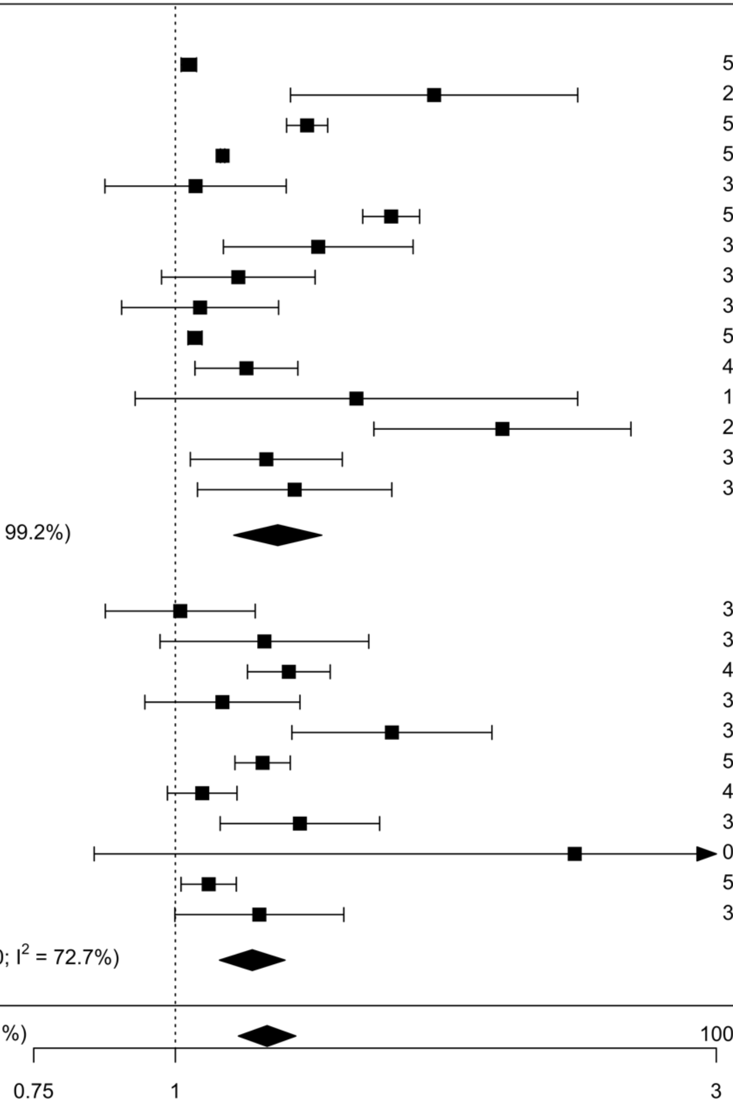

Supplementary Figure 6 Forest plot illustrating results from sensitivity analysis for the incident risk of all CVD in people with PD in studies that did not use a population of health professionals.

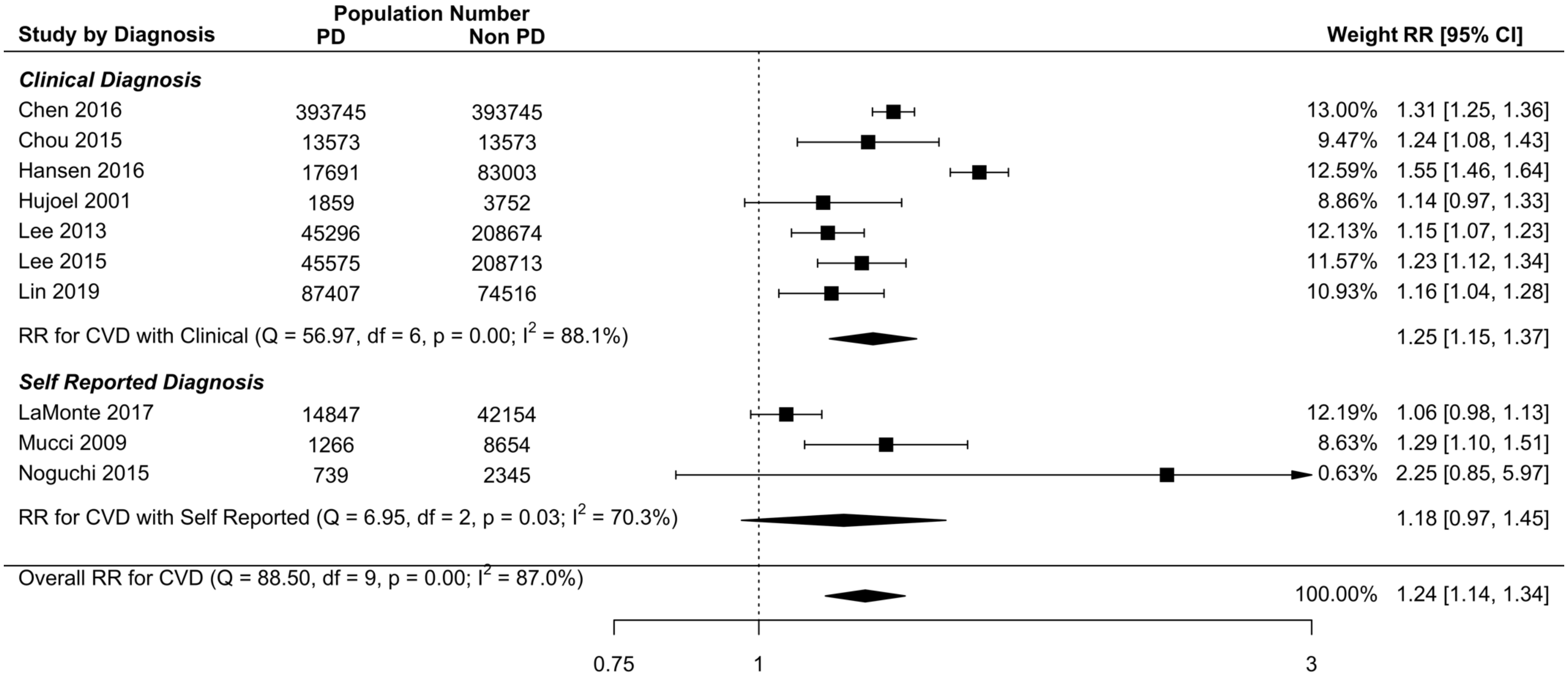

Supplementary Table 3 Summary table of excluded studies.

Study	Title	Reason for Exclusion
Chou 2015	Major adverse cardiovascular events in treated periodontitis: A population-based follow-up study from Taiwan	Duplicate
Chou 2018	Severity of chronic periodontitis and risk of gastrointestinal cancers: A population-based follow-up study from Taiwan	Duplicate
Demmer 2010	Periodontal Status and A1C Change	Incidence not calculated
Famili 2005	Longitudinal Study of Periodontal Disease and Edentulism With Rates of Bone Loss in Older Women	Incidence not calculated
Heikkila 2018	Periodontitis and cancer mortality: Register-based cohort study of 68,273 adults in 10-year follow-up	Incidence not calculated
Hung 2016	Allergic Rhinitis Is Associated With Periodontitis: A Population-Based Study	Incidence not calculated
Adrianne 2016	Role of periodontitis pathogens porphyromonas gingivalis and treponema denticola in the incidence of acute exacerbations of chronic obstructive pulmonary among indigenous elders on Madura Island	Ineligble publication format
Heianza 2018	Changes in dental health and coronary heart disease risk: Two prospective cohort studies in men and women	Ineligble publication format
Jiang 2013	A randomized controlled trial of pre-conception treatment for periodontal disease to improve periodontal status during pregnancy and birth outcomes	Ineligble publication format
Yang 2017	Tooth loss, liver cancer incidence, and chronic liver disease mortality in the ATBC study	Ineligble publication format
Han 2013	Correlation between periodontitis and chronic kidney disease in Korean adults	Ineligble study design
Lee 2015	Association of Lifestyle-Related Comorbidities With Periodontitis	Ineligble study design
Dizdar 2017	Increased cancer risk in patients with periodontitis	Ineligible control.
Heaton 2014	The influence of prevalent cohort bias in the association between periodontal disease progression and incident coronary heart disease	Ineligible control.
Holmlund 2017	Poor Response to Periodontal Treatment May Predict Future Cardiovascular Disease	Ineligible control.
Huang 2019	Association between intensive periodontal treatment and spontaneous intracerebral hemorrhage-a nationwide, population-based cohort study	Ineligible control.
Ide 2003	Effect of treatment of chronic periodontitis on levels of serum markers of acute-phase inflammatory and vascular responses	Ineligible control.
Jeffcoat 2003	Periodontal disease and preterm birth: results of a pilot intervention study	Ineligible control.
Kawabata 2016	Relationship Between Prehypertension/Hypertension and Periodontal Disease: A Prospective Cohort Study	Ineligible control.

Lee 2014	Surgical treatment for patients with periodontal disease reduces risk of end-stage renal disease: A nationwide population-based retrospective cohort study	Ineligible control.
Lin 2017	Association between periodontitis and the risk of palindromic rheumatism: A nationwide, population-based, case-control study	Ineligible control.
López 2002	Periodontal therapy may reduce the risk of preterm low birth weight in women with periodontal disease: a randomized controlled trial	Ineligible control.
Michalowicz 2009	Serum inflammatory mediators in pregnancy: changes after periodontal treatment and association with pregnancy outcomes	Ineligible control.
Morita 2010	A cohort study on the association between periodontal disease and the development of metabolic syndrome	Ineligible control.
Gomes 2016	Apical periodontitis and incident cardiovascular events in the Baltimore Longitudinal Study of Ageing	Ineligible study design.
Iwasaki 2015	Oral health status in relation to cognitive function among older Japanese	Ineligible study design.
Mendez 1998	An association between periodontal disease and peripheral vascular disease.	Ineligible study design.
Saito 2004	The severity of periodontal disease is associated with the development of glucose intolerance in non-diabetics: the Hisayama study	Ineligible study design.
Wood 2004	The relationship between tomato intake and congestive heart failure risk in periodontitis subjects	Ineligible study design.
Akinkugbe 2017	Periodontitis and Non-alcoholic Fatty Liver Disease, a population based cohort investigation in the Study of Health in Pomerania	Not CVD outcome
Arrive 2012	Oral health condition of French elderly and risk of dementia: a longitudinal cohort study	Not CVD outcome
Bertrand 2017	Periodontal disease and risk of non-Hodgkin lymphoma in the Health Professionals Follow-up Study	Not CVD outcome
Chang 2016	Investigating the Association Between Periodontal Disease and Risk of Pancreatic Cancer	Not CVD outcome
Choi 2017	Effect of periodontitis on the development of osteoporosis: results from a nationwide population-based cohort study (2003-2013).	Not CVD outcome
Chou 2015	Rheumatoid Arthritis Risk Associated with Periodontitis Exposure: A Nationwide, Population-Based Cohort Study.	Not CVD outcome
Chou 2018	Severity of chronic periodontitis and risk of gastrointestinal cancers: A population-based follow-up study from Taiwan.	Not CVD outcome
Chung 2016	A population-based study on the associations between chronic periodontitis and the risk of cancer	Not CVD outcome
Demmer 2008	Periodontal Disease and Incident Type 2 Diabetes	Not CVD outcome
Freudenheim 2016	Periodontal Disease and Breast Cancer: Prospective cohort of Postmenopausal Women	Not CVD outcome
Helenius-Hietala 2019	Periodontitis is associated with incident chronic liver disease-A population-based cohort study	Not CVD outcome
Hsu 2015	Association of Periodontitis and Subsequent Depression: A Nationwide Population-Based Study	Not CVD outcome
Hu 2018	Risk of colorectal cancer in patients with periodontal disease severity: a nationwide, population-based cohort study	Not CVD outcome

Iwasaki 2012	Periodontal Disease and Decreased Kidney Function in Japanese	Not CVD outcome
Kebede 2018	Does periodontitis affect diabetes incidence and haemoglobin A1c change? An 11-year follow-up study	Not CVD outcome
Keller 2012	The effects of chronic periodontitis and its treatment on the subsequent risk of psoriasis	Not CVD outcome
Lee 2017	Periodontal Disease Associated with Higher Risk of Dementia: Population-Based Cohort Study in Taiwan	Not CVD outcome
Lee 2017	Periodontitis as a Modifiable Risk Factor for Dementia: A Nationwide Population-Based Cohort Study	Not CVD outcome
Lin 2014	Association between periodontitis needing surgical treatment and subsequent diabetes risk: a population-based cohort study	Not CVD outcome
Lin 2018	Patients with chronic periodontitis present increased risk for primary Sjogren syndrome: A nationwide population-based cohort study	Not CVD outcome
Lin 2018	Increased Risk of Ulcerative Colitis in Patients with Periodontal Disease: A Nationwide Population-Based Cohort Study	Not CVD outcome
Lin 2019	Association between Periodontal Disease and Subsequent Sjogren's Syndrome: A Nationwide Population-Based Cohort Study	Not CVD outcome
Mau 2017	Patients with chronic periodontitis present increased risk for osteoporosis: A population-based cohort study in Taiwan	Not CVD outcome
Morita 2010	A cohort study on the association between periodontal disease and the development of metabolic syndrome.	Not CVD outcome
Miyawaki 2016	Self-reported periodontitis and incident type 2 diabetes among male workers from a 5-year follow-up to MY health up study	Not CVD outcome
Stein 2007	Tooth loss, dementia and neuropathology in the Nun Study	Not CVD outcome
Thistle 2018	Association of tooth loss with liver cancer incidence and chronic liver disease mortality in a rural Chinese population	Not CVD outcome
Wen 2014	Cancer risk among gingivitis and periodontitis patients: A nationwide cohort study	Not CVD outcome
Yang 2017	Tooth loss and liver cancer incidence in a Finnish cohort	Not CVD outcome
Arora 2009	An exploration of shared genetic risk factors between periodontal disease and cancers: a prospective co-twin study	Not CVD outcome
Soder 2011	Periodontal disease may associate with breast cancer	Not CVD outcome
Mai 2016	Periodontal disease severity and cancer risk in postmenopausal women: the Buffalo OsteoPerio Study	Not CVD outcome
Nwizu 2017	Periodontal Disease and Incident Cancer Risk among Postmenopausal Women: Results from the Women's Health Initiative Observational Cohort	Not CVD outcome
Michaud 2018	Periodontal Disease Assessed Using Clinical Dental Measurements and Cancer Risk in the ARIC Study	Not CVD outcome
Chen 2017	Association between chronic periodontitis and the risk of Alzheimer's disease: a retrospective, population-based, matched-cohort study.	Not CVD outcome
Demmer 2011	Periodontal disease, tooth loss and incident rheumatoid arthritis: results from the First National Health and Nutrition Examination Survey and its epidemiological follow-up study	Not CVD outcome

Arkema 2010	A Prospective cohort of periodontal disease and risk of rheumatoid arthritis	Not CVD outcome
Ide 2011	Periodontal disease and incident diabetes: a seven-year study	Not CVD outcome
Lin 2015	Association between periodontal disease and osteoporosis by gender: a nationwide population-based cohort study	Not CVD outcome
Nakib 2013	Periodontal disease and risk of psoriasis among nurses in the United States	Not CVD outcome
Michaud 2008	Periodontal disease, tooth loss, and cancer risk in male health professionals: a Prospective cohort	Not CVD outcome
Michaud 2007	A Prospective cohort of periodontal disease and pancreatic cancer in US male health professionals	Not CVD outcome
Takeuchi 2017	Tooth Loss and Risk of Dementia in the Community: the Hisayama Study	Not CVD outcome
Yamamoto 2012	Association between self-reported dental health status and onset of dementia: a 4-year Prospective cohort of older Japanese adults from the Aichi Gerontological Evaluation Study (AGES) Project	Not CVD outcome
Mai 2014	History of periodontal disease diagnosis and lung cancer incidence in the Women's Health Initiative Observational Study	Not CVD outcome
Barros 2013	A Cohort Study of the Impact of Tooth Loss and Periodontal Disease on Respiratory Events among COPD Subjects: Modulatory Role of Systemic Biomarkers of Inflammation	Pre-diagnosed systemic disease in population.
Beck 2008	The Periodontitis and Vascular Events(PAVE) Pilot Study: Adverse Events	Pre-diagnosed systemic disease in population.
Bresolin 2013	Lipid profiles and inflammatory markers after periodontal treatment in children with congenital heart disease and at risk for atherosclerosis	Pre-diagnosed systemic disease in population.
Chang 2017	Periodontal Pocket Depth, Hyperglycemia, and Progression of Chronic Kidney Disease: A Population-Based Longitudinal Study	Pre-diagnosed systemic disease in population.
Chen 2013	Association between a history of periodontitis and the risk of rheumatoid arthritis: a nationwide, population-based, case-control study	Pre-diagnosed systemic disease in population.
Han 2018	Oral Health Status and Behavior among Cancer Survivors in Korea Using Nationwide Survey	Pre-diagnosed systemic disease in population.
Hou 2017	Risk factors of periodontal disease in maintenance hemodialysis patients	Pre-diagnosed

		systemic disease in population.
Huang 2018	Intensive periodontal treatment reduces risks of hospitalization for cardiovascular disease and all-cause mortality in the hemodialysis population	Pre-diagnosed systemic disease in population.
Jamieson 2015	Periodontal disease and chronic kidney disease among Aboriginal adults; an RCT	Pre-diagnosed systemic disease in population.
Lee 2017	Association between periodontal disease and prostate cancer: results of a 12-year longitudinal cohort study in South Korea	Pre-diagnosed systemic disease in population.
Lertpimonchai 2019	Periodontitis as the risk factor of chronic kidney disease: Mediation analysis	Pre-diagnosed systemic disease in population.
Loesche 1998	Interactions between periodontal disease, medical diseases and immunity in the older individual	Pre-diagnosed systemic disease in population.
López 2012	Effects of periodontal therapy on systemic markers of inflammation in patients with metabolic syndrome: a controlled clinical trial	Pre-diagnosed systemic disease in population.
Mattila 1995	Dental infection and the risk of new coronary events: prospective study of patients with documented coronary artery disease	Pre-diagnosed systemic disease in population.
Shen 2016	Periodontal Treatment Reduces Risk of Adverse Respiratory Events in Patients with Chronic Obstructive Pulmonary Disease	Pre-diagnosed systemic disease in population.
Shen 2017	Impact of periodontal treatment on hospitalization for adverse respiratory events in asthmatic adults: A propensity-matched cohort study	Pre-diagnosed systemic disease in population.
Caúla 2014	The effect of periodontal therapy on cardiovascular risk markers: a 6-month randomized clinical trial.	Unclear outcome definition.

Earnshaw 1998	Tooth counts do not predict bone mineral density in early postmenopausal Caucasian women. EPIC study group	Unclear outcome definition.
Kaye 2010	Tooth loss and periodontal disease predict poor cognitive function in older men	Unclear outcome definition.
Singer 2018	The association of periodontal disease and cardiovascular disease risk: Results from the Hispanic Community Health Study/Study of Latinos	Unclear outcome definition.
Yoshihara 2007	Renal function and periodontal disease in elderly Japanese	Unclear outcome definition.
Bando 2017	Impact of oral self-care on incident functional disability in elderly Japanese: The Ohsaki Cohort 2006 study	Unclear PD definition
Krall 1996	Increased risk of tooth loss is related to bone loss at the whole body, hip, and spine	Unclear PD definition
